# Evolving Performance Management Systems in Public Sector Networks: A Longitudinal Case Study of The World Largest Public Network Supporting Smes

**DOI:** 10.12688/openreseurope.21281.1

**Published:** 2025-09-19

**Authors:** Stéphane Ruiz-Coupeau, Juan Manuel Ramón-Jerónimo, Raquel Florez-Lopez

**Affiliations:** 1 Department of Accounting and Financial Economics, University of Seville, Seville, Andalusia, 41004, Spain; 2 Department of Financial Economics and Accounting, Pablo de Olavide University, Seville, Andalusia, 41013, Spain

**Keywords:** Performance Management Systems evolution, Public sector networks, SMEs support organization

## Abstract

**Background:**

Performance management systems (PMS) in public sector networks face unique challenges due to distributed governance, heterogeneous actors, and evolving policy priorities. While collaborative networks are increasingly central to policy implementation, little is known about how PMS evolve in such settings. This paper investigates the longitudinal evolution of the Enterprise Europe Network (EEN)—the world’s largest public network supporting small and medium enterprises (SMEs)—over fifteen years, offering new insights into PMS as socio-technical systems.

**Methods:**

The study employs a qualitative, longitudinal single-case design, using abductive reasoning. Data collection combined extensive archival analysis (calls for proposals, grant agreements, evaluation reports, coordination artefacts) with direct observation of network routines and governance arenas. Coding was conducted in iterative stages—open, axial, and selective—to identify recurrent dynamics, triggers of PMS change, and building blocks of system evolution.

**Results:**

Findings reveal that the EEN’s PMS evolved through five phases, shifting from activity/output-based reporting to a client-journey logic that captures SME achievements, impact, and cross-consortia contributions. Evolution occurred in episodic cycles rather than continuous adaptation, with contractual boundaries fixing indicators within multiannual programmes. Redesign was triggered by external forces and enacted through governance arenas. A generic three-layer framework is developed, comprising contextual triggers, lifecycle phases, and building blocks.

**Conclusions:**

The study demonstrates that PMS in public sector networks evolve as socio-technical systems shaped by external triggers, bounded lifecycles, and building blocks. This reframes PMS not as static indicator sets nor as continuously adaptive systems, but as episodic, governed design processes that balance accountability, collaboration, and learning. The proposed framework is transferable to other public sector networks and provides actionable guidance for policymakers and managers seeking to design performance systems that capture the value of coordination and co-production in complex, multi-actor environments.

## Plain Language Summary

Public sector networks supporting small and medium enterprises bring together many organisations across countries to provide advice, training, and partnership opportunities. However, measuring how well such large and complex networks perform is not straightforward. Traditional performance systems that work in single organisations are not sufficient when outcomes depend on the collaboration of many partners. This study looks at the world’s largest public network supporting SMEs, active in more than 40 countries for over 15 years. Using official documents and direct observations, we traced how the network’s performance management system changed over time. We found that it evolved from simply counting activities (like events and contacts) to following SMEs along their “client journey”, tracking achievements and long-term business impacts such as new partnerships, innovation, and market growth. The research shows that these systems do not change continuously but in cycles, usually linked to EU policy and funding periods. Each cycle brings redesigns based on evaluations, new tools, or stakeholder feedback. We developed a framework to explain this process.

## 1. Introduction

Traditional performance measurement designed for single organisations or programmes struggle to capture the effectiveness of collaborative public networks ( Heranz, 2010; Wang & Ran, 2021). In settings where multiple, semi-autonomous actors must collaborate to produce outcomes, network managers are still held accountable for results even though they lack the hierarchical authority assumed by conventional public management tools ( Lee, 2022). This accountability–authority gap generates two problems at once: a coordination dilemma on how to steer distributed actions and a methodological dilemma: how to attribute performance in the presence of interdependence and joint production ( Lægreid & Rykkja, 2022). While research on collaborative governance shows that networks can outperform siloed approaches in complex policy arenas, we still know relatively little about how performance is defined, measured and *managed* inside multi-actor networks over time, and how measurement systems themselves evolve as networks mature and missions evolve ( Agranoff, 2004; Agranoff & McGuire, 2001; Bouwer *et al.*, 2021; Heranz, 2010; Koppenjan, 2008; Provan & Milward, 2001; Provan *et al.*, 2007; Wang & Ran, 2021).

Evaluating networked service delivery is intrinsically harder than assessing single-agency programmes. Client or user pathways are non-linear and often bespoke; value is co-produced across organisational boundaries; and outcomes emerge from sequences of services rather than from one network member’s discrete intervention. Network performance sits at the intersection of technical, institutional and political complexity ( Klijn *et al.*, 2025); the same performance information may be interpreted differently by actors with divergent preferences, mandates or risk appetites. In such contexts, *what counts as success* must be negotiated, not just measured, implying that performance management systems (PMS) in networks are socio-technical systems that must evolve to remain both legitimate and useful ( Karina *et al.*, 2025; Trist, 1981).

The increasing prominence of networks implementing public policies stems from their potential to address complex societal challenges more effectively than traditional bureaucratic structures ( Agranoff & McGuire, 2001; Kickert *et al.*, 1997; Siciliano *et al.*, 2021). However, the mechanisms through which collaborative efforts translate into performance and how PMS can be designed to support and reflect that process remain insufficiently understood ( Costumato, 2021; Provan *et al.*, 2007).

This paper addresses these gaps by focusing on design and the evolution of PMS in public networks dedicated to SME support, a domain marked by multi-level governance, substantive complexity, and shifting policy priorities. The Enterprise Europe Network (EEN) ^1^ -the world’s largest public network supporting SMEs- provides an ideal longitudinal case for this investigation, having operated across more than 40 countries for over 15 years Its relevance is underscored by the European Commission’s strategic commitment to positioning SMEs at the core of its vision for a sustainable, digital, and resilient economy, given that SMEs represent 99% of all EU businesses and account for two-thirds of private sector employment ^2^. They are essential drivers of innovation, competitiveness, and local development, yet face numerous challenges in accessing finance, entering new markets, and adapting to green and digital transitions.

The EU has responded to these challenges through a series of policy strategies—including the 2020 SME Strategy ^3^, the Industrial Strategy ^4^, and the 2023 SME Relief Package ^5^—that emphasize the role of business support networks like the EEN. These initiatives aim to reduce administrative burdens, facilitate access to investment, and improve SME access to services and cross-border opportunities. The EEN provides tailored advisory services, partner search support, and training to thousands of SMEs annually. Its scale, decentralized governance, and evolving mission make it a compelling setting for examining PMS evolution in practice.

In such contexts, designing and operating effective PMS is inherently challenging. Yet PMS remain essential for coordinating action, fostering accountability, and supporting learning across fragmented systems ( Deschamps & Mattijs, 2018). Recognizing PMS as evolving socio-technical systems rather than fixed sets of indicators is key to understanding their role in complex public networks ( Karina *et al.*, 2025; Trist, 1981).

Despite the growing scholarly interest in performance management within networked forms of governance, important gaps persist in both theory and practice ( Waardenburg *et al.*, 2025). One of the most significant limitations is the tendency to conceptualize the PMS as static sets of indicators or reporting tools. Much of the literature focuses on PMS as fixed frameworks designed to monitor outputs and outcomes, paying little attention to how these systems change, adapt, or evolve over time ( Cosa & Torelli, 2024). This static view overlooks the dynamic nature of governance networks and the need for PMS to respond to shifting strategies, stakeholder configurations, and contextual developments ( Braz *et al.*, 2011; Kennerley & Neely, 2003).

A further limitation is the lack of empirical application of PMS theory to transnational public networks. While there is a well-established body of literature on PMS in local or national public organizations ( van der Kolk, 2022), relatively few studies have examined performance management in publicly financed programmes such as the Enterprise Europe Network (EEN). These networks are unique in their multi-level governance structures, combining central EU-level coordination with decentralized implementation across diverse national and regional contexts. As a result, performance measurement in such settings requires tailored approaches that reflect both the complexity of cross-border coordination and the diversity of implementation practices on the ground.

This study seeks to address these gaps by developing both a conceptual and an empirical understanding of the evolution of PMS within public sector networks, and consequently poses the following research question:

*How do performance management systems evolve within public sector networks?*

The study uses a qualitative, longitudinal single-case design centred on the EEN’s PMS. We triangulate extensive archival analysis (calls for proposals, grant agreements, indicator dictionaries, evaluation reports and coordination artefacts over 15 years) with direct observation of network routines (e.g., thematic groups, representative bodies, and time-bounded working groups tasked with PMS/KPI redesign). Analytically, we combine temporal bracketing to trace how the PMS moved from activity/output counting to a client-journey logic with verifiable achievements and impact, complemented by contribution metrics (for cross-partner production) and quality ratios (for efficiency and effectiveness). Later, with inductive coding and abductive research logic we build a transferable design-and-evolution framework for PMS in public networks.

The paper contributes to scholarship and practice in two ways. Conceptually, it reframes network performance management as an iterative, governed design process rather than a one-off technical exercise, specifying the triggers (policy cycles, evaluations, IT shifts, stakeholder signals), lifecycle phases (design, use, adjustment, consolidation) and building blocks (journey–achievement–impact logic, contribution metrics, quality ratios, data pipeline, and governance arenas) that enable evolution. Empirically, it offers the first longitudinal account of PMS change in large trans-national public network for SME support. Practically, it distils actionable guidance for policymakers and network managers on how to measure the coordination that creates value, not only end outcomes, without sacrificing comparability across a large, heterogeneous system.

The remainder of the paper is structured as follows. Section 2 develops the theoretical framework, reviewing existing models of PMS evolution and highlighting their limitations when applied to public sector networks. Section 3 presents the methodology, outlining the longitudinal case study design, data sources, and abductive analytical strategy. Section 4 reports the empirical findings, first tracing the evolution of the EEN’s PMS across successive cycles and then presenting the generic framework derived from coding and abductive reasoning. Section 5 discusses the theoretical contributions, policy and managerial implications, and avenues for future research. Finally, Section 6 concludes the paper.

## 2. Theoretical framework

Understanding how and why performance management systems (PMS) evolve in public sector networks requires grounding in the broader transformations that have taken place in public administration and governance. Over the past four decades, public sector management has shifted from traditional hierarchical models to more decentralized, interactive, and collaborative forms of governance. These changes have reshaped not only how public services are delivered but also how performance is conceptualized, measured, and managed ( Gunn *et al.*, 2021; Klijn *et al.*, 2025).

Performance measurement is generally defined as the process of assessing the efficiency and effectiveness of past actions ( Neely *et al.*, 1995). Within this context, PMS refers to a coordinated set of performance indicators or metrics used to interpret and understand the performance of an organizational entity ( Carlsson-Wall *et al.*, 2016). At their core, PMS are information systems that transform input data into actionable performance metrics, enabling managers to assess progress, provide feedback, and steer future actions ( Burney & Matherly, 2007). These systems also support the implementation of strategy, process control, communication, and the articulation of organizational priorities ( Kaplan & Norton, 2000; Wouters & Sportel, 2005).

In the public sector, PMS are designed to enhance efficiency and effectiveness across public programmes and institutions. While primarily intended to support rational decision-making, they may also serve symbolic or ritualistic purposes ( Pollitt, 2013). PMS typically rely on decision-relevant performance indicators, often benchmarked against internal standards or external norms ( Blalock, 1999), to inform systematic reporting to administrative and political stakeholders ( Poister, 2008). The purposes served by these systems are multiple and interrelated, ranging from accountability, learning, and policy revision to planning and operational control ( Behn, 2003; Deschamps & Mattijs, 2018). Importantly, PMS data is also used for evaluation purposes, though a distinction is made between ongoing performance management and periodic evaluations that assess the broader relevance and impact of policies ( Perrin, 1998).

The evolution of public performance management cannot be understood without considering the wider paradigmatic shifts in public sector governance. The traditional public administration (TPA) model, dominant throughout much of the twentieth century, relied on formal hierarchies, standardized procedures, and rule-bound bureaucracy to ensure neutrality and accountability in service delivery ( Hughes, 2017; Osborne, 2006). However, this model became increasingly inadequate in the face of complex, multi-sectoral challenges and the growing scale of public service demands, what Rose (1981) described as the “Big Government” problem. Emerging in response, the New Public Management (NPM) paradigm introduced private-sector management techniques to the public domain. It promoted output-based accountability, market mechanisms, performance contracts, and customer orientation. Public managers were encouraged to adopt tools such as benchmarking and performance indicators, enabling them to “steer” rather than “row” ( Osborne & Gaebler, 1992). While NPM contributed significantly to the institutionalization of PMS, it also introduced fragmentation and often overemphasized measurable outcomes at the expense of broader public value or collective governance goals. To address the limitations of both TPA and NPM, a third paradigm, the "network governance", gained prominence. This approach recognizes that many policy problems are “wicked” and cannot be solved by single organizations acting in isolation. Instead, they require coordinated efforts among interdependent actors from public, private, and nonprofit sectors ( Klijn *et al.*, 2025; Koppenjan *et al.*, 2019). Governance networks are marked by horizontal coordination, shared decision-making, and mutual interdependence. In such settings, traditional hierarchical control mechanisms become less effective, and performance cannot be solely attributed to individual actors. This presents unique challenges for the design and evolution of PMS, which must now account for multiple institutional logics, diverse stakeholder interests, and distributed accountability ( Vikstedt & Vakkuri, 2025).

The literature on network governance draws from several traditions, each emphasizing different facets of networked interaction. The policy networks tradition examines the influence of interest groups on public policymaking; the service delivery tradition focuses on coordination challenges in fragmented service systems; and the managing networks tradition explores how complex policy problems are addressed through inter-organizational collaboration ( Kickert *et al.*, 1997). Despite their differences, these traditions converge around the idea that outcomes are co-produced by multiple actors, and that performance is the emergent property of interactions rather than the result of any single organization’s actions.

Supporting SMEs through public networks constitutes a complex governance challenge ( Knox & Arshed, 2022; Pulka *et al.*, 2021). Problems in this domain are rarely technical or clearly defined. Instead, they are substantively complex, marked by competing definitions of problems and diverse interpretations of success ( Klijn *et al.*, 2025). Actors in these networks like national governments, regional agencies, business support organizations, and SMEs themselves, often hold divergent views on priorities, solutions, and desirable outcomes. Moreover, wicked problems, such as internationalization, innovation diffusion, and sustainability, cut across economic, social, and regulatory domains ( Benito & Meyer, 2024). These issues are not only difficult to solve, but also difficult to measure. Performance information does not always reduce uncertainty, and can sometimes exacerbate disagreement, as actors interpret the same data in different ways ( Baekgaard & Serritzlew, 2015). In addition, public debates and political discourse can influence how performance is framed, perceived, and acted upon ( Baekgaard *et al.*, 2019).

From an organizational theory perspective, networks are understood to emerge when organizations require resources controlled by others and engage in sustained interaction to access them ( Levine & White, 1961; Rogers & Whetten, 1982). These interactions are especially relevant in the delivery of complex services and are governed by coordination mechanisms that range from formal to informal ( Park *et al.*, 2021). As such, network effectiveness depends not only on structure but also on the nature of coordination ( Hjern & Porter, 1981; Raab *et al.*, 2015). Indeed, at the heart of networked governance lies the challenge of aligning heterogeneous coordination logics. Public agencies often operate under bureaucratic logics emphasizing control, accountability, and compliance; private-sector actors tend to prioritize flexibility, competition, and innovation; and nonprofit organizations typically rely on trust, shared values, and informal norms. Herranz (2007), Herranz (2010) synthesizes these into three ideal coordination orientations: bureaucratic, entrepreneurial, and community-based. Managing these conflicting logics involves balancing consistency, adaptability, and collaboration, a dilemma he calls the “management trilemma.” Each orientation implies distinct understandings of performance and demands different design features in the PMS. Therefore, network managers must not only coordinate actions but also reconcile competing rationalities within the performance system itself.

Another critical element lies in the insufficient integration of complexity into existing PMS models. Collaborative networks—particularly those involved in SME support—operate under conditions of substantive, strategic, and institutional complexity ( Hovik & Hanssen, 2015). Substantive complexity arises from divergent perceptions among actors regarding goals, priorities, and the very definition of success. Strategic complexity reflects the shifting interests and power dynamics within and across network participants, while institutional complexity stems from varying legal, administrative, and cultural frameworks across jurisdictions. Yet, much of the existing PMS literature assumes a level of stability and uniformity that does not reflect these realities. As Klijn *et al.* (2025) argue, PMS in governance networks must contend with these multifaceted challenges, but most models have yet to adequately account for them.

In this complex environment, the design of PMS requires more than the technical selection of indicators. Logic models have emerged as useful heuristic tools for aligning strategy with measurement. A logic model articulates the hypothesized causal pathway from inputs and activities to outputs and outcomes. It facilitates stakeholder engagement, clarifies assumptions, and supports the planning, monitoring, and evaluation of public programmes ( Frechtling, 2025; Hatry, 2006; Julian, 1997; Kaplan & Garrett, 2005). Because logic models are collaboratively developed, they serve as boundary objects that bridge diverse perspectives, making them especially well-suited for public networks where agreement on objectives and success criteria must be negotiated. Herranz, Jr. (2010) adapted logic models for use in network settings emphasizing their capacity to integrate performance measurement with collaborative governance. In these contexts, logic models offer structure without rigidity and can accommodate dynamic changes in strategy or coordination arrangements. They also help address a key gap in the literature: the disconnect between network coordination processes and measurable outcomes ( Koppenjan, 2008). By foregrounding causality, shared goals, and measurable results, logic models support the design of PMS that are not only technically robust but also politically and operationally feasible in networked contexts.

While logic models provide an entry point for PMS design, they do not fully explain how PMS evolve over time. For this, scholars have proposed conceptual frameworks such as the performance management life-cycle model ( Gutierrez *et al.*, 2015; van Helden *et al.*, 2012) in which PMS development typically follows four interrelated stages: design, implementation, use, and assessment. In the design phase, objectives and indicators are defined in alignment with strategy. Implementation involves system rollout and integration into organizational routines. The use phase encompasses the application of performance data for decision-making, learning, and control. Finally, the assessment phase evaluates the effectiveness of the system and may lead to redesign. Importantly, this cycle is iterative rather than linear; organizations learn from each stage and adjust accordingly through feedback and feedforward loops.

Complementing this view, Kennerley and Neely ( 2002; 2003) and later Hald and Mouritsen (2018) propose a four-phase evolutionary model of PMS change: use, reflection, modification, and deployment. These models emphasize that PMS evolution is often triggered by internal misalignments, such as gaps between performance measures and strategic priorities, or external shocks like policy reforms or stakeholder pressure. They highlight that PMS change is not merely a technical adjustment but a social and political process, often marked by resistance, negotiation, and institutional constraints. In public sector networks, where authority and control are distributed, PMS change is particularly challenging.

Additionally, the literature from operations management and management accounting further reinforces that PMS must remain dynamic and adaptable to their environment ( Bourne *et al.*, 2000; Busco *et al.*, 2007; Cosa & Torelli, 2024; Gutierrez *et al.*, 2015). Evolution may be deliberate, as in formal redesign processes, or emergent, resulting from informal reinterpretations or the gradual drift of measurement practices over time. Scholars have highlighted the role of informal systems, organizational routines, and learning processes in shaping the effectiveness and trajectory of PMS ( Braz *et al.*, 2011; Deschamps & Mattijs, 2018; Wouters & Sportel, 2005). From an institutional perspective, PMS are socially constructed systems whose meaning and relevance are constantly negotiated across actors and contexts ( Andon *et al.*, 2007; Quattrone & Hopper, 2001; van de Ven & Poole, 1995).

These models fall short when applied to public sector networks, which are characterised by distributed governance and multiple institutional layers. In these contexts, PMS development emerges through negotiation among diverse actors with often competing priorities, making the process externally shaped, co-produced, and subject to continuous adjustment rather than linear progression. Moreover, these models underplay the role of exogenous triggers which frequently redefine measurement priorities and indicators. Unlike traditional models that frame change as a product of internal learning, PMS evolution in public networks is often reactive and shaped by external inputs ( Agostino & Arnaboldi, 2018). The process is also non-linear and iterative, involving ongoing adjustments to indicators and metrics in response to operational frictions and shifting goals. In addition, the coexistence of conflicting performance logics across governance levels and the lack of attention to infrastructural supports further complicate PMS evolution in networks ( Vikstedt & Vakkuri, 2025). These dynamics underscore the need for new conceptual tools that go beyond single-organisation assumptions to capture the complex, negotiated nature of PMS change in public sector networks.

Taken together, these theoretical perspectives provide a rich foundation for analyzing the evolution of PMS in public sector networks. The transition from hierarchical government to networked governance, the interplay of diverse coordination logics, the potential of logic models for structuring performance expectations, and the dynamic, iterative nature of PMS development point to the need for nuanced, context-sensitive approaches. This study applies these lenses to a longitudinal case of a large public network, using an abductive approach to build a framework that integrates the theoretical insights discussed above with the empirical realities of performance management in public networks.

## 3. Methodology

This research adopts a qualitative, longitudinal single-case study design to examine the evolution of the PMS within the EEN — a European network that supports SMEs. The study focuses on how performance indicators, measurement tools, governance arrangements, and managerial practices evolve over time in response to institutional pressures, policy priorities, and learning dynamics within a multi-level, trans-national network structure.

The case study approach is justified given the exploratory nature of the research question, which seeks to understand how PMSs are constructed and transformed over an extended time horizon. Following Yin (2018), the single-case design is appropriate when the case is critical, revelatory, and longitudinal, offering a rare opportunity to observe organizational phenomena as they evolve in real time within their natural context. The EEN case is uniquely suitable given its temporal depth (2008–2025), pan-European structure, public governance logic, and exposure to formalized performance regimes from the outset.

The research is grounded in an abductive research logic. Abduction allows the researcher to incorporate unexpected observations and practical reasoning into theory-building, especially suitable for institutional and processual research domains like PMS development. This epistemological orientation also supports a practice-based view of PMSs ( Nicolini *et al.*, 2016), not just as technical tools, but as evolving artefacts embedded in socio-material routines, institutional logics, and political dynamics ( Jansson *et al.*, 2020).

### Case design and units of analysis

The EEN constitutes a single, coherent European network of regional business-support organisations (almost 600 network members) operating across all EU regions and associated countries under the Single Market Programme ^6^. It is co-financed by the European Commission through multi-annual grants and is mandated to help SMEs innovate, grow, and internationalise, while contributing to EU objectives on competitiveness, sustainability, digitalisation, resilience, and entrepreneurship. The network works through a client-centric logic, integrating first-level information services and second-level, tailored advisory and partnering services that are expected to generate measurable business impact for SMEs. Its governance includes EU-level coordination, a representative body, and an architecture of Sector and Thematic Groups that enable knowledge exchange and joint service delivery across consortia.

EEN is theoretically and empirically suited for a study of PMS evolution for three reasons. First, it is the world’s largest public network supporting SMEs, offering a rich setting to observe how PMS accommodate multi-actor coordination, EU-level steering, and local delivery. Second, the programme spans multiple cycles providing temporal depth to trace evolution. This temporal structuring allows the research to trace institutional layering and shifts in performance logic over time. Third, the lead researcher’s extended professional experience within the network afforded access to archives and meetings that are typically difficult to observe, while also necessitating explicit reflexive strategies to mitigate role-related bias. Indeed, to address potential bias associated with this position, we employed (i) source triangulation (archives, administrative data, observations), (ii) a structured audit trail linking claims to documents and dated events, and (iii) reflexive memoing to separate descriptive notes from interpretive commentary. No personal or commercially sensitive information is reported; evidence is presented in aggregate or anonymised form consistent with organisational guidelines.

The empirical focus is the EEN ‘s PMS, with data collected from the CESEAND consortium ^7^ (the Andalusian node of EEN) and its engagement with EU-level structures.

### Data collection

The research uses multiple data sources in line with case study best practices. Data were collected over several years through document analysis, direct observation, and memo writing, enabling both retrospective and real-time insights into the evolving PMS.

The empirical material is classified into four main categories, as summarized in Table 1 below:

**Table 1.** Underlying data sources used in the study.

Category	Document Title	Code
**EU Call for** ** proposals**	Call for proposals CELEX_C2006_306_07_EN	1.1
	Terms of reference EEN 2017–2018_en	1.2
	Call for proposals_smp-cosme-2021-een_en	1.3
	Call for proposals_smp-cosme-2024-een_en	1.4
**Evaluation** **reports**	Evaluation report_eip_en 2011	2.1
	Evaluation report_eaci_2011_en	2.2
	Evaluation report_EEN_2008–2014	2.3
**Memos**	Memo PMS used by the EEN period 2008–2025	3.1
	Memo CESEAND Plus Period 2015–2016	3.2
	Memo CESEAND Plus Period 2019–21	3.3
	Memo Working Group on Measurement and KPIs	3.4
	Memo Training Improve Your Achievements	3.5
	Memo Thematic Group Start-up and Scale-up	3.6
**Complementary** ** sources**	Conference proceedings on EEN (2008–2020)	4.1
	Report EEN Our Story 2008–2020	4.2

The calls for proposals provide insight into the formal expectations and design logic underpinning each programming cycle. The external evaluation reports, authored by independent contractors on behalf of the European Commission, offer a meta-assessment of the EEN’s performance effectiveness, relevance, and coherence with policy objectives. The researcher memos and field notes — developed between 2015 and 2023 — are based on participation in internal meetings, working groups, training sessions, and archival analysis, including grants agreements and project reporting. These constitute primary ethnographic style data, capturing practices, conversations, concerns, and interpretations among EEN members. Finally, the complementary sources help contextualize the Network’s institutional narrative and learning trajectory. All these documents are included as underlying data ( Ruiz-Coupeau, 2025), ensuring transparency and traceability of interpretations.

### Data analysis

The data analysis proceeded in two distinct but complementary stages.

The first stage aimed at describing the evolution of the PMS across different phases. To achieve this, we employed temporal bracketing ( Langley, 1999), a processual analysis technique that structures longitudinal data into successive periods separated by identifiable shifts. Temporal bracketing allows the researcher to capture both continuity and change by segmenting a process into analytically meaningful intervals, while preserving the temporal ordering of events. This method is particularly suitable for studying evolving organizational systems, as it highlights how earlier configurations create the conditions and constraints for subsequent developments.

In practice, we reconstructed a timeline of the EEN’s PMS by bracketing the material into programming phases (2008–2012; 2012–2015; 2015–2020; 2021–2025; and a forward-looking phase beyond 2025). Within each bracket, we organized archival documents and observation notes to identify key design decisions, governance arrangements, data infrastructure shifts, and revisions to indicators/KPIs and quality processes. This structuring yielded a longitudinal narrative of PMS evolution that made visible the episodic, cyclical character of change, while also clarifying how triggers and building blocks carried over across successive funding periods.

The second stage moved beyond description to develop a conceptual framework explaining the observed evolution. Here, the coding procedure combined systematic qualitative coding with an abductive logic of inquiry. Following Peirce (1934) conception, abduction refers to the process of moving from empirical observations and existing theory to the generation of plausible explanatory models creating new theory. This approach was particularly suited to our aim of reconstructing the evolution of the EEN’s PMS and developing a more generalizable framework for PMS design and evolution in public sector networks ( Pfister *et al.*, 2023).

The first stage of analysis involved *open coding*, where each document was read iteratively, and relevant passages were coded using descriptive terms. Codes captured three broad domains: performance artefacts (e.g. indicators, dashboards, reporting formats, databases); practices and routines (e.g. peer learning, mentoring, knowledge sharing, training); and governance mechanisms (e.g. working groups, steering committees, evaluation protocols). This process generated a comprehensive inventory of elements that constituted the PMS.

In the second stage, *axial coding* was used to cluster related codes into higher-order categories that expressed the mechanisms of PMS evolution. For instance, “EU calls,” “grant agreements,” and “policy cycles” were grouped under the category of policy and funding triggers. Likewise, “external audits,” “evaluation recommendations,” and “impact indicators” were consolidated into a category representing evaluations as redesign triggers. Finally, “client journey,” “achievements,” and “impact pathways” were grouped into a category of measurement logic. Axial coding thus enabled the identification of recurrent dynamics linking codes across sources and over time.

The final stage, *selective coding*, connected these axial categories into a coherent explanatory model. Here, abductive reasoning was central: we sought the most plausible structure to integrate the empirical findings with theoretical insights on performance systems, organizational learning, and network governance. This process led to the construction of a generic framework for the design and evolution of PMS in public networks, which integrates three interconnected layers.

Through this abductive process, the framework was not “discovered” inductively as given, but constructed as the most plausible explanatory account of PMS evolution. It is grounded in the detailed coding of EEN documents and observations, yet abstracted to a level that renders it transferable to other public sector networks. Thus, abduction provided a bridge between empirical richness and theoretical generalisation, strengthening both the explanatory power and practical relevance of the framework.

## 4. Findings

These findings are presented in two steps: (1) the evolution of PMS across the different phases, and (2) the construction of a generic framework for the design and evolution of PMS in public networks:

### 4.1. Evolution of PMS in 15 years

The findings are structured around distinct phases in the evolution of the EEN (2008–2012; 2012–2015; 2015–2020; 2021–2025; and a forward-looking phase beyond 2025). These phases were identified by the researcher based on shifts in PMS structures, with each period characterized by relative stability before subsequent changes emerged. The analysis indicates that the PMS did not develop along a linear trajectory of technical refinement, but rather evolved through cyclical patterns.

Across five phases, the EEN’s PMS shifted from an activity/output-oriented dashboard to a networked, client-journey architecture that tracks achievements and impact. The early list of indicators suite primarily counted events, contacts and hours; subsequent cycles introduced logic-model elements and, later, a formal client-journey structure linking advanced advisory and partnering services to measurable achievements and business impact for SMEs. In the most recent phase, a consolidated set of key performance indicators (KPIs) and quality ratios embeds network logics (e.g., contributions across consortia) and emphasises coordination, capacity building and quality management.

***Phase 1 (2008–2012): activity and output orientation.*** In the first phase, services were organised under three broad objectives—information/feedback and internationalisation, innovation and technology/knowledge transfer, and support to participate in Research and Technological Development (RTD) projects —consistent with the Competitiveness and Innovation Programme (CIP) ^8^. Monitoring focused on activity volumes and immediate outputs. For instance, EEN members reported counts of events, media outputs (press articles, newsletters), brokerage activity, and time allocations, with limited capture of downstream effects. These indicators demonstrate a strong emphasis on promotion, service reach and transactional brokerage metrics in the early PMS design.

The external evaluation covering the period highlighted high client satisfaction and European added value, while calling for clearer vision/leadership, stronger visibility, and “more impact-oriented monitoring”. It also noted the introduction of a new IT system whose integrative capacity had yet to be assessed and observed that policy feedback functions required substantial effort with uncertain regulatory impact, points that foreshadowed later PMS redesigns.

**Figure 1.**
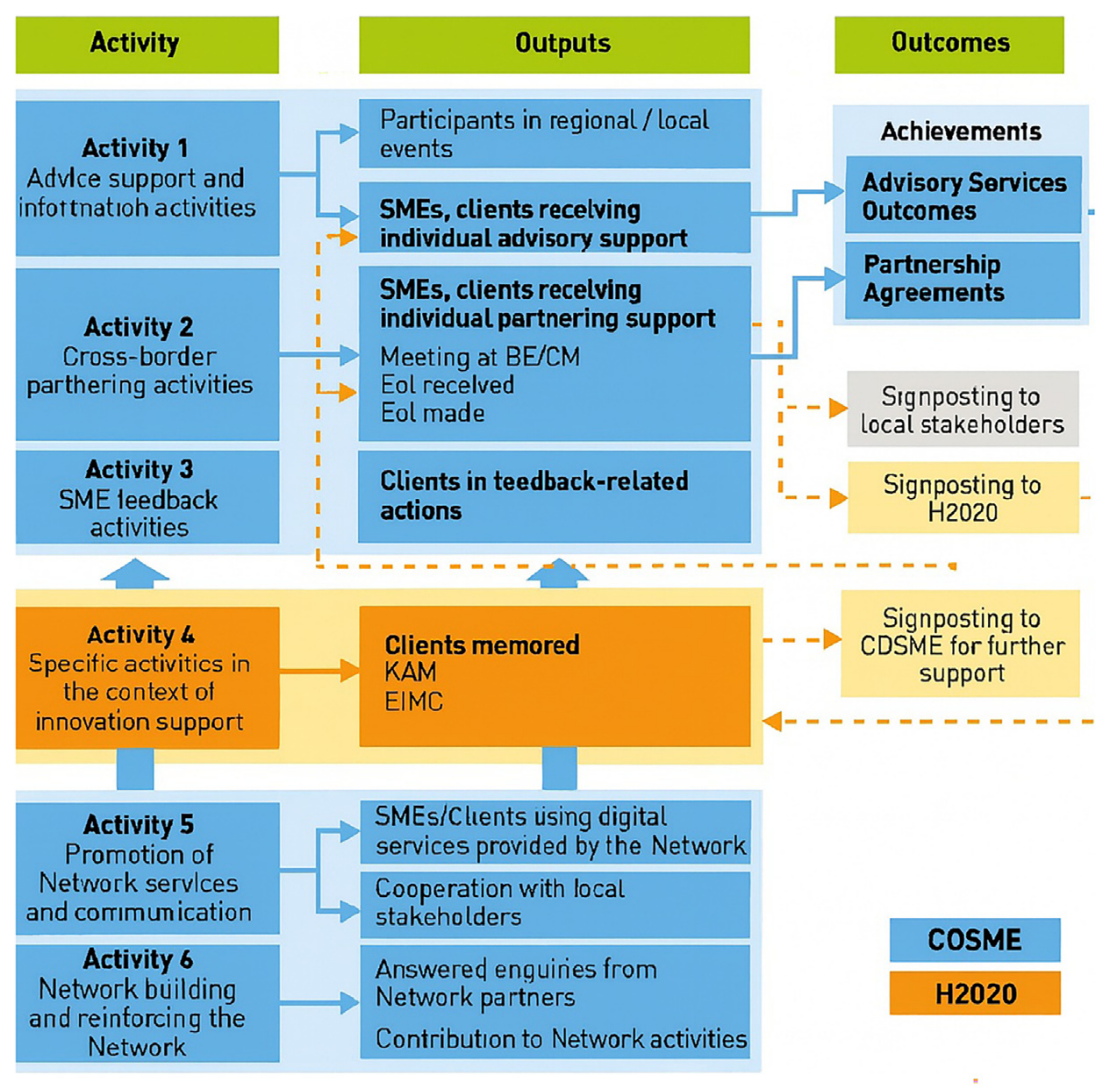
EEN activities, outputs and outcomes. Retrieved from document “1.2 Terms of reference EEN 2017-2018_en” Ruiz-Coupeau (2025).

***Phase 2 (2012–2015): consolidation and preparation for redesign.*** The second phase consolidated service families and deepened innovation and partnering activities, but the monitoring system remained largely outcome-centric while groundwork was laid for a more explicit logic-model approach as can be seen in Figure 1 where activities, output and outcomes are graphically displayed.

Three of the six standard activities of the Network can be understood through a logic model approach, where each activity is designed to generate specific outputs that ultimately lead to outcomes (SME achievements). Advisory, support, and information services (activity 1) provide SMEs with direct guidance on EU policies and programs, producing outputs such as informed businesses and tailored advice, which in turn strengthen SMEs’ competitiveness and innovation capacity (Advisory service outcomes). Cross-border cooperation activities (activity 2) generate outputs like business meetings, which translate into outcomes such partnerships, technology transfer agreements, and research collaborations (Partnership agreements). Similarly, innovation support activities (activity 4) deliver outputs such as diagnostic reports and action plans for SMEs, along with coaching connections through the Key Account Manager system; the outcome is stronger innovation management and higher success in European R&D initiatives (Advisory service outcomes).

Besides, network governance arrangements (e.g., EU-level coordination with a representative body) began to formalise communication and change-management channels, setting the stage for more coordinated PMS development across countries and consortia.

***Phase 3 (2015–2020): introduction of the client-journey.*** A major step-change occurred with the explicit adoption of a client-journey logic that sequences first-level (basic) and second-level (advanced) services. Second-level services, either advisory or partnering, were now expected to culminate in Achievements (milestones) and, ultimately, business impact for SMEs (e.g., increase in market share, turnover, cost optimisation/savings, jobs, quality and innovation outcomes). This recast the PMS from counting isolated interactions to tracing cumulative value over time, aligning service delivery with measurable milestones and impact categories.

Concurrently, network learning infrastructures matured. Sector and Thematic Groups expanded their role as competence centres, supporting advanced casework and disseminating methods and tools across consortia—an enabling condition for consistent measurement and comparable “achievement” definitions.

**Figure 2.**
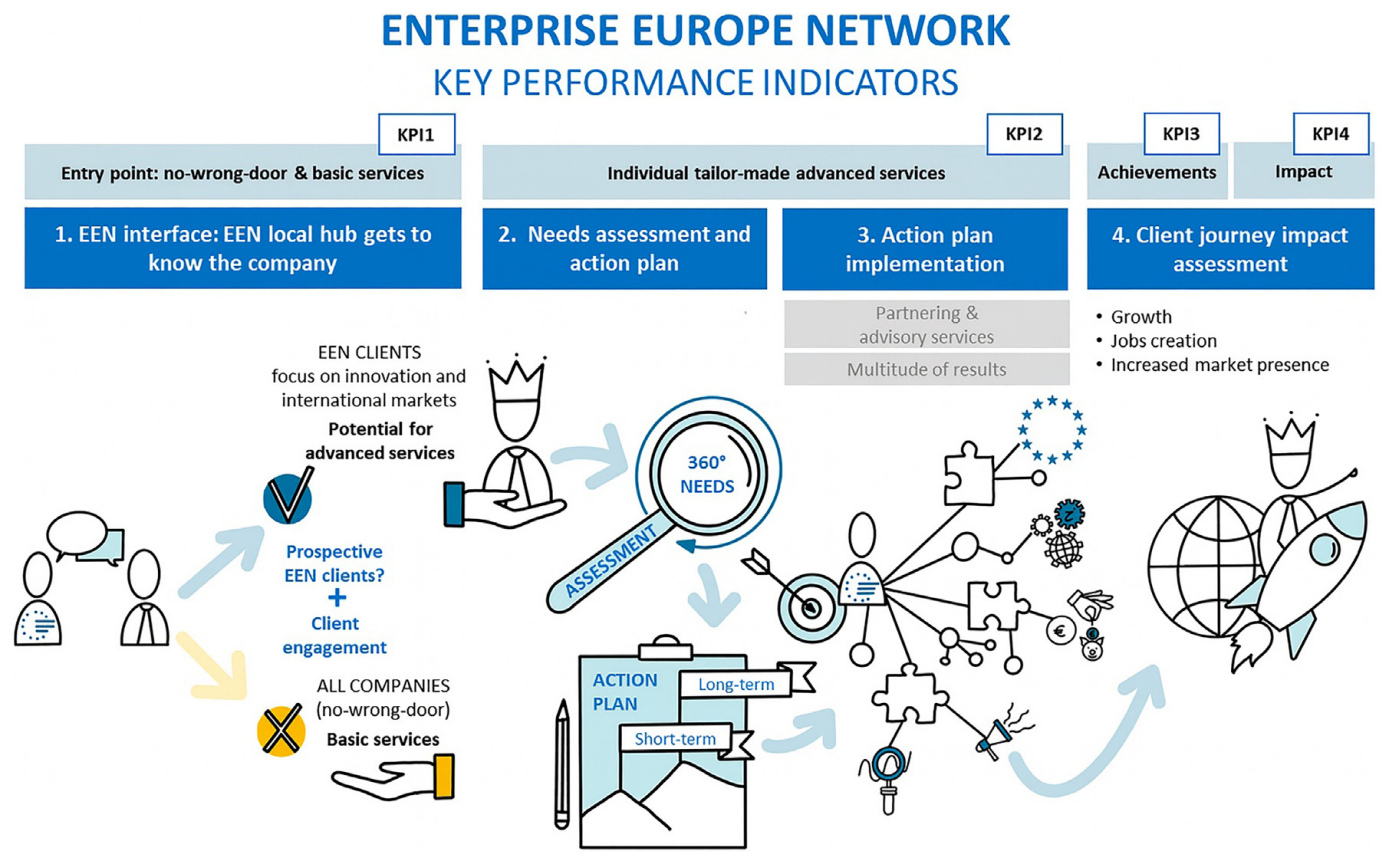
Client journey and corresponding KPIs. Source ( European commission, 2021).

***Phase 4 (2021–2025): KPI consolidation, quality ratios and network logics.*** Under the Single Market Programme, activities were streamlined into four pillars: value-added services to clients, promotion/communication, network development & capacity building, and coordination & quality management. The PMS is consolidated integrating KPI suite (KPI1–KPI4) focused in the client journey as shown in Figure 2:

Four quality ratios completed the client-journey architecture and embedded network coordination. Quality ratios track efficiency and effectiveness (e.g., achievements per client, clients reporting impact per unique client in the client journey, achievements per FTE, average impact per client), sharpening attention to value creation rather than volumes alone. New metrics include unique clients in the client journey (KPI2) and impact on clients (KPI4). Activity indicators capturing contribution metrics are included: contribution to other partners’ journeys and network development activities. SME feedback is also included as an activity indicator.

Governance mechanisms were strengthened to support this shift. A representative body (plenary and bureau) acts as a sounding board, relays network views to the Commission and Agency, and leads the implementation of changes across countries; Sector and Thematic Groups function as hubs for expert exchange, common approaches and learning, including feedback on Single Market obstacles or disruptions. Importantly, temporary working groups were convened with a limited mandate to redesign the PMS and KPIs, evidencing a deliberate, participatory change process.

***Phase 5 (2025 →): stabilising the model and extending project management.*** The next cycle retains the four activity pillars and explicitly adds project management as a fifth activity for all consortia, signalling institutionalisation of coordination and quality routines alongside service delivery. The continuity of the KPI suite and quality ratios indicates consolidation of the client-journey measurement model, with an emphasis on cross-consortia contributions and capacity building as levers of network-level performance.

Taken together, the EEN case shows a managed yet non-linear evolution of PMS: external accountability demands, and internal learning pressures periodically align to produce redesigns; governance forums translate these pressures into shared definitions and routines; and the measurement focus progressively shifts from outputs to journey-anchored achievements and impact, with explicit metrics for networked production and quality. The result is a PMS that better fits the realities of collaborative SME support while retaining comparability and steerability across a large, heterogeneous transnational network.

### 4.2. Construction of a generic framework for the evolution of PMS in public networks

The generic framework constructed through an abductive process, weaving together archival evidence (calls for proposals, grant agreements, evaluations, Commission communications) with direct observations of the EEN at both European and consortium level. Abduction was crucial because neither a purely deductive application of theory nor an inductive generalisation from observations could fully account for the distinctive dynamics of PMS in large, contractual public networks. Instead, by iteratively moving between theory and data, we inferred a model that explains both the rigidity of KPIs within funding cycles and the recurrent, structured evolution of the PMS across cycles.

The coding of documents and memos revealed recurrent patterns that were grouped into higher-order elements. These ranged from external forces, such as policy cycles or evaluation pressures, to internal processes of PMS development, and to the technical and institutional components that stabilise measurement practices.

Table 2 illustrates the analytical path from open coding to the final framework. The first four rows group codes relating to *contextual triggers*. These highlight how PMS in public networks is shaped by forces external to the network itself. The next four rows show how these triggers interact with the *PMS lifecycle*, which we conceptualise as a phase-bounded cycle of *design, appropriation, evaluation, and renewal*. The final five rows describe the *building blocks* at the core of the PMS. Taken together, the table shows the outcome of a grounded coding process. Each element can be traced back to specific archival categories and observed practices in the EEN, and their organisation into outer triggers, lifecycle phases, and inner building blocks reflects the layered nature of PMS evolution in public networks. The results of the coding are summarised in Table 2, which demonstrates the progression from empirical descriptors (open codes) to analytical clusters (axial categories) and selective codes.

**Table 2.** From open coding to framework elements.

Open coding categories	Framework element (axial categories)	Framework layer (selective codes)
Multiannual programmes, calls, grant agreements	Policy & funding cycles	Contextual triggers
External evaluations, recommendations	Evaluations	Contextual triggers
CRM/reporting portals, templates, evidence protocols	IT & reporting changes	Contextual triggers
SME surveys, partner feedback, EC/EISMEA guidance	Stakeholder feedback	Contextual triggers
Call texts and grant negotiation of KPI set	Design	PMS lifecycle
Training, thematic groups, mentoring, QA routines	Appropriation	PMS lifecycle
Mid/final evaluations, review meetings, WG debates	Evaluation	PMS lifecycle
WG synthesis, drafting of new indicators for next call	Renewal	PMS lifecycle
Client-journey logic (journey → achievements → impact)	Measurement logic	Building blocks
Cross-partner assists, shared achievements	Contribution metrics	Building blocks
Ratios/standards (achievements per client, FTE, impact share)	Quality lens	Building blocks
Definitions, evidence rules, metadata, CRM	Data pipeline	Building blocks
Representative body, thematic groups, KPI design WGs	Governance arenas	Building blocks

***Abductive construction of the framework.*** The movement from the coded categories in Table 2 to the layered framework is made possible by abductive reasoning. Abduction differs from deduction and induction: it is not a linear application of theory to data, nor a simple generalisation from observations, but a creative process of inference to the “best possible explanation” of observed patterns. In our case, archival material and direct observation produced numerous categories—policy cycles, evaluations, IT infrastructures, stakeholder feedback, grant specifications, internal trainings, governance bodies, measurement logics, quality ratios, and more. At first glance, these categories seemed heterogeneous and unconnected.

Through abductive analysis, we asked what kind of organizing logic could plausibly integrate our observations into a coherent model of PMS evolution. Iterative coding and comparison revealed two striking empirical regularities. First, performance indicators were never modified within a funding period but only at its contractual boundaries, which challenges models that assume continuous adjustment of measures. Second, despite this formal fixity, the network was far from static: actors were constantly appropriating the system, reflecting on its adequacy, and preparing proposals for future redesign. These patterns could not be fully explained by conventional PMS models that emphasize incremental change and ongoing recalibration of indicators. Abduction therefore required us to reframe the cycle not as continuous evolution but as a sequence of phases—design, appropriation, evaluation, and renewal—punctuated by external contractual boundaries.

A second abductive move concerned the clustering of categories. Categories such as policy cycles, evaluations, IT upgrades, and stakeholder feedback clearly referred to forces external to the day-to-day operation of the PMS. Interpreting them as contextual triggers allowed us to explain why system redesign happens episodically. Categories such as KPI specification, trainings, evaluations, and renewal debates described the temporal unfolding of the PMS; reinterpreting them as a lifecycle of phases gave coherence to the EEN’s recurrent rhythm. Finally, categories such as measurement logic, contribution metrics, quality ratios, data pipeline, and governance arenas cut across cycles and triggers; abductively, it made sense to treat them as building blocks, the stable elements that make the system function at any point in time.

Thus, the abductive process did not merely sort categories but actively searched for the most plausible explanatory configuration. The result is a three-layer generic framework: contextual triggers as the outer drivers of change, the PMS lifecycle as the patterned rhythm of design and use, and the building blocks as the core architecture. Each layer gains meaning only in relation to the others: without triggers, there would be no renewal; without building blocks, appropriation would not be possible; without the lifecycle, triggers and blocks would remain unconnected. The framework therefore represents not just a classification but an abductively reasoned explanation of how PMS in public networks evolve over time.

**Figure 3.**
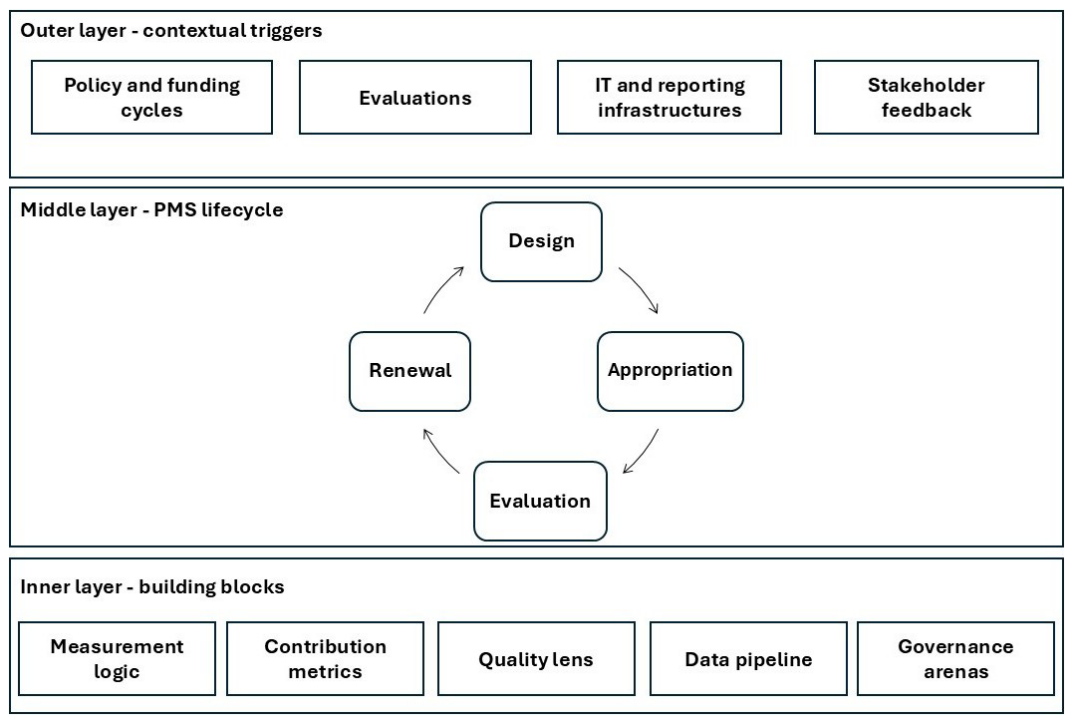
Generic framework for the evolution of PMS in public networks.

The abductive moves described above provided the interpretive logic that transformed the coded categories of Table 2 into the three-layered framework represented in Figure 3:

This framework provides a transferable model for understanding PMS evolution in any type of public sector network:

***Outer layer: contextual tiggers.*** At the outermost level, PMS evolution in public networks is driven by contextual triggers that set both the opportunities and constraints for change. These include *policy and funding cycles*, which define priorities, allocate resources, and establish the formal architecture of performance systems. *Evaluations* introduce legitimacy pressures, identifying shortcomings and recommending reforms that may be taken up in subsequent cycles. *IT and reporting infrastructures* provide the technical means through which data are collected, validated, and transmitted, and shifts in these systems can alter what is measurable and comparable. Finally, *stakeholder feedback* (from service users, frontline organisations, oversight bodies, and funding agencies) supplies the continuous input necessary to question whether the PMS remains meaningful and proportionate. In any public sector network, these triggers act as external forces that periodically compel performance systems to adapt.

***Middle layer: the PMS lifecycle.*** Within these triggers, the PMS evolves through a patterned lifecycle that repeats across policy or funding cycles. The lifecycle does not involve continuous adjustment of indicators, but rather a phase-bounded process structured around four stages:

1. *Design*: At the beginning of a cycle, indicators, targets, and reporting obligations are specified, often through formal agreements, contracts, or regulations.
2. *Appropriation*: Once fixed, the PMS is enacted and internalised by network members. This involves training, capacity-building, peer learning, and the development of shared interpretations to ensure consistent application across diverse organisations.
3. *Evaluation*: Over time, the PMS is tested through monitoring, external reviews, and after-action assessments, generating evidence about its feasibility, credibility, and utility.
4. *Renewal*: At the end of the cycle, insights from evaluation and stakeholder feedback are synthesised into proposals for the next round of design. Renewal thus represents the moment when the PMS can be reshaped, but always within the context of new policy and funding priorities.

This lifecycle is transferable because most public sector networks operate under time-bound mandates or funding arrangements that naturally segment PMS development into distinct cycles, rather than allowing for continuous adjustment.

***Inner layer: building blocks.*** At the core of the framework are the building blocks that provide the technical and institutional foundation of the PMS. The *measurement logic* defines how the system conceptualises performance, for example by linking activities to outputs, outcomes, and broader impacts. *Contribution metrics* recognise that in networked settings, results are often co-produced and attribution is shared among multiple actors. The *quality lens* introduces comparability and legitimacy, typically through ratios, benchmarks, or minimum standards. The *data pipeline* encompasses the definitions, evidence rules, IT systems, and audit protocols that ensure credibility and reproducibility of performance information. Finally, *governance arenas* (committees, working groups, or representative bodies) are the spaces where PMS are interpreted, contested, and redesigned. These building blocks are generic in the sense that any public sector network requires a performance logic, a way to attribute contributions, mechanisms for quality, reliable data infrastructures, and institutional settings for governance.

***Integration of layers.*** The explanatory power of the framework lies in the interaction of the three layers. Contextual triggers generate the impetus for change, but they are translated into concrete adaptations only through the lifecycle, where design, appropriation, evaluation, and renewal unfold in sequence. The building blocks provide the stable components that enable each phase to function and give continuity across cycles. This configuration makes the framework transferable: regardless of policy domain, scale, or geography, public sector networks evolve their PMS by responding to contextual pressures, following bounded lifecycles, and relying on core building blocks.

This framework explains why PMS in networks are stable within funding cycles but evolve across them, and how technical building blocks interact with governance arenas to ensure both continuity and adaptability. More broadly, the framework demonstrates that PMS in networks should not be conceptualised as static indicator systems nor as continuously adaptable tools, but as episodic, socio-technical systems that balance contractual stability with responsiveness to external triggers and internal learning. By offering this layered and cyclical view, our study extends existing theory and opens new avenues for comparative research across domains such as health, education, innovation, and environmental governance.

## 5. Discussion

This study set out to answer the question: *How do performance management systems (PMS) evolve within public sector networks?* Drawing on a longitudinal case study of the Enterprise Europe Network (EEN), our findings indicate that PMS in such networks evolve through episodic cycles rather than continuous adjustment, with contractual boundaries delimiting periods of stability and opportunities for redesign. The generic three-layer framework developed—comprising contextual triggers, lifecycle phases, and building blocks—helps explain these dynamics and provides a transferable lens for examining PMS evolution across different policy domains.

This study contributes to the literature on PMS evolution in three interrelated ways, each of which extends and refines existing theoretical perspectives.

First, we reframe PMS evolution as episodic and contractually bounded. Much of the PMS literature has conceptualised performance systems as capable of continuous adaptation, with measures incrementally updated in response to strategic or operational shifts ( Bourne *et al.*, 2000; Braz *et al.*, 2011; Kennerley & Neely, 2002; Kennerley & Neely, 2003; Wouters & Sportel, 2005). This perspective is consistent with intra-organisational settings where managers retain discretion to recalibrate indicators in line with strategy. Our findings, however, show that in public sector networks such as the EEN, PMS are locked into multi-annual funding frameworks, with indicators fixed for the duration of contractual periods. Redesign occurs only at contractual boundaries, triggered by external evaluations, policy reforms, or IT infrastructure changes ( Agostino & Arnaboldi, 2018). This supports recent calls to pay greater attention to the temporal and institutional structuring of PMS evolution ( Waardenburg *et al.*, 2025) and suggests that models which assume linear, incremental change ( Hald & Mouritsen, 2018; van Helden *et al.*, 2012) must be extended to incorporate the episodic nature of networked governance systems.

Second, the study advances the institutional theory of PMS by emphasising networks as arenas of negotiated meaning. Prior research shows that PMS are not neutral tools but rather embody different institutional logics ( Andon *et al.*, 2007; Carlsson-Wall *et al.*, 2016; Quattrone & Hopper, 2001). Yet, few studies have examined how this plays out in transnational, multi-actor networks. In the EEN, PMS design and use required the reconciliation of bureaucratic logics of control, entrepreneurial logics of innovation, and community logics of trust and reciprocity ( Herranz, 2007; Heranz, 2010). Governance arenas, such as representative bodies, thematic groups, and time-bound KPI working groups, played a central role in mediating contestation and stabilising shared definitions of success. This resonates with Provan and Milward’s (2001) emphasis on negotiation in network performance, but also extends it by showing how PMS themselves become boundary objects ( Heranz, 2010; Millar *et al.*, 2001) that accommodate institutional diversity while ensuring comparability. The finding also connects to Vikstedt and Vakkuri (2025), who highlight the coexistence of multiple logics in collaborative PMS, and to Koppenjan (2008), who stresses that success in networks is always negotiated rather than objectively defined.

Third, the findings deepen socio-technical perspectives on PMS by foregrounding the role of infrastructures. PMS evolution is not solely a matter of indicator design or governance negotiation but also of infrastructural supports. Building on socio-technical systems theory ( Trist, 1981) and recent insights into digitalisation and big data integration in PMS ( Cosa & Torelli, 2024; Karina *et al.*, 2025), our analysis shows that CRM systems, reporting templates, metadata protocols, and audit procedures actively shape what counts as measurable and legitimate performance. In the EEN, the introduction of new IT systems or evidence protocols redefined both the scope of measurement and the comparability of results across consortia. This aligns with Moynihan’s (2008) argument that infrastructures not only enable performance information flows but also structure decision-making, and with Deschamps and Mattijs (2018), who underline the role of PMS in supporting organisational learning. By highlighting the infrastructural dimension, our study adds to recent work that urges scholars to view PMS as socio-technical artefacts embedded in political and institutional contexts ( Jansson *et al.*, 2020; Vikstedt & Vakkuri, 2025).

Taken together, these contributions extend theorisations of PMS in three directions: (i) by embedding temporality through episodic, contractually bounded cycles of change; (ii) by situating PMS as arenas of negotiated meaning where multiple institutional logics are reconciled; and (iii) by recognising infrastructural supports as constitutive elements of PMS evolution. This integrated perspective enriches performance management theory by moving beyond static models of indicator design and beyond purely technical accounts of system change, offering instead a socio-technical, institutional, and temporal understanding of how PMS evolve in public sector networks. Implications for policy and management

### Implications for policy and management

For policymakers, the study demonstrates that PMS design in networks should be recognised as a policy instrument in itself, shaping not only accountability but also collaboration and learning. The European Commission’s experience with the EEN suggests that mandating outcome-oriented KPIs is necessary but insufficient: equal attention must be given to creating governance arenas and data infrastructures that allow diverse actors to internalise, interpret, and use indicators consistently.

For network managers, the findings imply that performance management should be treated as a continuous design process rather than a compliance exercise. Managers should invest in appropriation mechanisms like training, thematic groups or peer learning, to ensure that PMS becomes a shared practice rather than a formal reporting burden. The introduction of contribution metrics and quality ratios in the EEN offers a practical template for rewarding collaboration and efficiency in multi-actor settings.

More broadly, the framework provides guidance for designing PMS in other public supported networks (e.g., environmental, health and education programmes) where balancing comparability with contextual diversity remains a pressing challenge.

### Limitations and venues for future research

Several limitations should be acknowledged. First, the study is based on a single-case design. While the EEN represents a revelatory case, further comparative research across different policy domains (e.g., health, environment, education) is needed to test the transferability of the proposed framework.

Second, the data sources rely heavily on archival and observational material from within the EEN. Although triangulation and reflexive strategies were applied, future research could complement this with SME-level perspectives on how performance indicators influence service uptake and perceived value.

Third, the study focuses on the formal PMS as codified in EU calls, evaluations, and reporting tools. Informal practices of sensemaking, negotiation, and local adaptation deserve closer ethnographic attention to capture how PMS are enacted “on the ground” ( Andon *et al.*, 2007).

Future research could therefore pursue three directions: (i) cross-case comparisons of PMS evolution in different transnational networks; (ii) mixed-methods studies that integrate performance data with qualitative accounts of user experiences, including informal practices. The third direction could be the analysis of digitalisation and big-data integration in network PMS, extending socio-technical perspectives to account for emerging data sources and analytics ( Cosa & Torelli, 2024; Karina *et al.*, 2025).

## 6. Conclusion

This paper examined how PMS evolve in public sector networks through a longitudinal case study of the EEN. By tracing fifteen years of PMS design, appropriation, evaluation, and renewal, it showed that evolution is not linear or continuous but episodic and contractually bounded, shaped by contextual triggers and enacted through governance arenas.

The proposed three-layer framework — contextual triggers, lifecycle phases, and building blocks — offers a transferable model for understanding PMS evolution in networks. It demonstrates that PMS should be seen as socio-technical systems that balance stability and adaptability, integrating measurement logic, contribution metrics, quality lenses, data infrastructures, and governance arenas.

The findings contribute to theory by embedding temporal, institutional, and infrastructural dimensions into PMS research, and to practice by providing actionable lessons for policymakers and network managers seeking to design performance systems that foster both accountability and collaboration.

Ultimately, by recognising PMS as evolving design processes rather than static indicator sets, public sector networks can better capture the value of collaborative action and support SMEs — and other beneficiaries — in addressing Europe’s most pressing economic and societal challenges.

## Ethics and consent

This study is based on archival materials and researcher memos. The memos draw on the researcher’s participation in internal meetings, working groups, and training sessions within the Enterprise Europe Network. No personal data, sensitive information, or commercially confidential material are included in this study. All evidence is presented in aggregate or anonymised form, in line with organisational guidelines and ethical best practices. As the research relies on documentary sources and non-identifiable observational material, it did not require formal ethical approval or written informed consent from individual participants.

## Data Availability

Zenodo: Underlying data - PMS of the EEN ( https://doi.org/10.5281/zenodo.16911874) Ruiz-Coupeau (2025). This research contains the following underlying data: Call for proposals CELEX_C2006_306_07_EN Terms of reference EEN 2017–2018_en Call for proposals_smp-cosme-2021-een_en Call for proposals_smp-cosme-2024-een_en Evaluation report_eip_en 2011 Evaluation report_eaci_2011_en Evaluation report_EEN_2008–2014 Memo PMS used by the EEN period 2008–2025 Memo CESEAND Plus Period 2015–2016 Memo CESEAND Plus Period 2019–21 Memo Working Group on Measurement and KPIs Memo Training Improve Your Achievements Memo Thematic Group Start-up and Scale-up Conference proceedings on EEN (2008–2020) Report EEN Our Story 2008–2020 Data are available under the terms of the Creative Commons Attribution 4.0 International license (CC-BY 4.0)

## Footnotes

^1^ Enterprise Europe Network https://een.ec.europa.eu/

^2^ SME Performance Review 2023, https://single-market-economy.ec.europa.eu/smes/sme-strategy/smeperformance-review_en

^3^ SME Strategy for a sustainable and digital Europe, COM(2020) 103 final.

^4^ A New Industrial Strategy for Europe, COM(2020) 102 final.

^5^ SME Relief Package, COM(2023) 535

^6^ European Commission’s Single Market Programme https://commission.europa.eu/funding-tenders/find-funding/eu-funding-programmes/single-market-programme/overview_en

^7^ CESEAND – Enterprise Europe Network node of the region of Andalucia (Spain) https://een-andalucia.es/

^8^ European Commision’s Competitiveness and Innovation Programme https://ec.europa.eu/cip/

## Figure permissions

The authors have created and own Figure 3. Figure 1 and Figure 2 were obtained from EU Funding & Tenders Portal. And as per the European Commission’s publications rules, these are licensed under the Creative Commons Attribution 4.0 International (CC BY 4.0) licence, which implies that they can be reused, if the source is acknowledged. That has been done in this manuscript. More information could be found here https://commission.europa.eu/legal-notice_en#copyright-notice
