# Evolving Performance Management Systems in Public Sector Networks: A Longitudinal Case Study of The World Largest Public Network Supporting Smes

**DOI:** 10.12688/openreseurope.21281.2

**Published:** 2026-01-08

**Authors:** Stéphane Ruiz-Coupeau, Juan Manuel Ramón-Jerónimo, Raquel Florez-Lopez

**Affiliations:** 1 Department of Accounting and Financial Economics, University of Seville, Seville, Andalusia, 41004, Spain; 2 Department of Financial Economics and Accounting, Pablo de Olavide University, Seville, Andalusia, 41013, Spain

**Keywords:** Performance Management Systems evolution, Public sector networks, SMEs support organization

## Abstract

**Background:**

Performance management systems (PMS) in public sector networks face unique challenges due to distributed governance, heterogeneous actors, and evolving policy priorities. While collaborative networks are increasingly central to policy implementation, little is known about how PMS evolve in such settings. This paper investigates the longitudinal evolution of the Enterprise Europe Network (EEN), the world’s largest public network supporting small and medium enterprises (SMEs), over fifteen years, offering new insights into PMS as socio-technical systems.

**Methods:**

The study employs a qualitative, longitudinal single-case design, using abductive reasoning. Data collection combined extensive archival analysis (calls for proposals, grant agreements, evaluation reports, coordination artefacts) with direct observation of network routines and governance arenas. Coding was conducted in iterative stages-open, axial, and selective-to identify recurrent dynamics, triggers of PMS change, and building blocks of system evolution.

**Results:**

Findings reveal that the EEN’s PMS evolved through five phases, shifting from activity/output-based reporting to a client-journey logic that captures SME achievements, impact, and cross-consortia contributions. Evolution occurred in episodic cycles rather than continuous adaptation, with contractual boundaries fixing indicators within multiannual programmes. Redesign was triggered by external forces and enacted through governance arenas. A generic three-layer framework is developed, comprising contextual triggers, lifecycle phases, and building blocks.

**Conclusions:**

The study demonstrates that PMS in public sector networks evolve as socio-technical systems shaped by external triggers, bounded lifecycles, and building blocks. This reframes PMS not as static indicator sets nor as continuously adaptive systems, but as episodic, governed design processes that balance accountability, collaboration, and learning. The proposed framework is transferable to other public sector networks and provides actionable guidance for policymakers and managers seeking to design performance systems that capture the value of coordination and co-production in complex, multi-actor environments.

## Plain Language Summary

Public sector networks supporting small and medium enterprises bring together many organisations across countries to provide advice, training, and partnership opportunities. However, measuring how well such large and complex networks perform is not straightforward. Traditional performance systems that work in single organisations are not sufficient when outcomes depend on the collaboration of many partners. This study looks at the world’s largest public network supporting SMEs, active in more than 40 countries for over 15 years. Using official documents and direct observations, we traced how the network’s performance management system changed over time. We found that it evolved from simply counting activities (like events and contacts) to following SMEs along their “client journey”, tracking achievements and long-term business impacts such as new partnerships, innovation, and market growth. The research shows that these systems do not change continuously but in cycles, usually linked to EU policy and funding periods. Each cycle brings redesigns based on evaluations, new tools, or stakeholder feedback. We developed a framework to explain this process.

## 1. Introduction

Traditional performance measurement designed for single organisations or programmes struggle to capture the effectiveness of collaborative public networks ( Heranz, 2010; Wang & Ran, 2021). In settings where multiple, semi-autonomous actors must collaborate to produce outcomes, network managers are still held accountable for results even though they lack the hierarchical authority assumed by conventional public management tools ( Lee, 2022). This accountability–authority gap generates two problems at once: a coordination dilemma on how to steer distributed actions and a methodological dilemma: how to attribute performance in the presence of interdependence and joint production ( Lægreid & Rykkja, 2022). While research on collaborative governance shows that networks can outperform siloed approaches in complex policy arenas, we still know relatively little about how performance is defined, measured and *managed* inside multi-actor networks over time, and how measurement systems themselves evolve as networks mature and missions evolve ( Agranoff, 2004; Agranoff & McGuire, 2001; Bouwer *et al.*, 2021; Heranz, 2010; Koppenjan, 2008; Provan & Milward, 2001; Provan *et al.*, 2007; Wang & Ran, 2021).

Evaluating networked service delivery is intrinsically harder than assessing single-agency programmes. Client or user pathways are non-linear and often bespoke; value is co-produced across organisational boundaries; and outcomes emerge from sequences of services rather than from one network member’s discrete intervention. Network performance sits at the intersection of technical, institutional and political complexity ( Klijn *et al.*, 2025); the same performance information may be interpreted differently by actors with divergent preferences, mandates or risk appetites. In such contexts, *what counts as success* must be negotiated, not just measured, implying that performance management systems (PMS) in networks are socio-technical systems that must evolve to remain both legitimate and useful ( Karina *et al.*, 2025; Trist, 1981).

The increasing prominence of networks implementing public policies stems from their potential to address complex societal challenges more effectively than traditional bureaucratic structures ( Agranoff & McGuire, 2001; Kickert *et al.*, 1997; Siciliano *et al.*, 2021). However, the mechanisms through which collaborative efforts translate into performance and how PMS can be designed to support and reflect that process remain insufficiently understood ( Costumato, 2021; Provan *et al.*, 2007).

This paper addresses these gaps by focusing on design and the evolution of PMS in public networks dedicated to SME support, a domain marked by multi-level governance, substantive complexity, and shifting policy priorities. The Enterprise Europe Network (EEN) ^1^ -the world’s largest public network supporting SMEs- provides an ideal longitudinal case for this investigation, having operated across more than 40 countries for over 15 years Its relevance is underscored by the European Commission’s strategic commitment to positioning SMEs at the core of its vision for a sustainable, digital, and resilient economy, given that SMEs represent 99% of all EU businesses and account for two-thirds of private sector employment ^2^. They are essential drivers of innovation, competitiveness, and local development, yet face numerous challenges in accessing finance, entering new markets, and adapting to green and digital transitions.

The EU has responded to these challenges through a series of policy strategies—including the 2020 SME Strategy ^3^, the Industrial Strategy ^4^, and the 2023 SME Relief Package ^5^ that emphasize the role of business support networks like the EEN. These initiatives aim to reduce administrative burdens, facilitate access to investment, and improve SME access to services and cross-border opportunities. The EEN provides tailored advisory services, partner search support, and training to thousands of SMEs annually. Its scale, decentralized governance, and evolving mission make it a compelling setting for examining PMS evolution in practice.

In such contexts, designing and operating effective PMS is inherently challenging. Yet PMS remain essential for coordinating action, fostering accountability, and supporting learning across fragmented systems ( Deschamps & Mattijs, 2018). Recognizing PMS as evolving socio-technical systems rather than fixed sets of indicators is key to understanding their role in complex public networks ( Karina *et al.*, 2025; Trist, 1981).

Despite the growing scholarly interest in performance management within networked forms of governance, important gaps persist in both theory and practice ( Waardenburg *et al.*, 2025). One of the most significant limitations is the tendency to conceptualize the PMS as static sets of indicators or reporting tools. Much of the literature focuses on PMS as fixed frameworks designed to monitor outputs and outcomes, paying little attention to how these systems change, adapt, or evolve over time ( Cosa & Torelli, 2024). This static view overlooks the dynamic nature of governance networks and the need for PMS to respond to shifting strategies, stakeholder configurations, and contextual developments ( Braz *et al.*, 2011; Kennerley & Neely, 2003).

A further limitation is the lack of empirical application of PMS theory to transnational public networks. While there is a well-established body of literature on PMS in local or national public organizations ( van der Kolk, 2022), relatively few studies have examined performance management in publicly financed programmes such as the Enterprise Europe Network (EEN). These networks are unique in their multi-level governance structures, combining central EU-level coordination with decentralized implementation across diverse national and regional contexts. As a result, performance measurement in such settings requires tailored approaches that reflect both the complexity of cross-border coordination and the diversity of implementation practices on the ground.

This study seeks to address these gaps by developing both a conceptual and an empirical understanding of the evolution of PMS within public sector networks, and consequently poses the following research question:

*How do performance management systems evolve within public sector networks?*

Although descriptive in form, this research question is appropriate for an abductive and exploratory design, where the aim is to reconstruct the mechanisms by which PMS evolve rather than to test predefined hypotheses. The three-layer framework developed in this study operationalises the question by linking contextual triggers, lifecycle phases and building blocks of PMS evolution in public networks.

The study uses a qualitative, longitudinal single-case design centred on the EEN’s PMS. We triangulate extensive archival analysis (calls for proposals, grant agreements, indicator dictionaries, evaluation reports and coordination artefacts over 15 years) with direct observation of network routines (e.g., thematic groups, representative bodies, and time-bounded working groups tasked with PMS/KPI redesign). Analytically, we combine temporal bracketing to trace how the PMS moved from activity/output counting to a client-journey logic with verifiable achievements and impact, complemented by contribution metrics (for cross-partner production) and ratios (for efficiency and effectiveness). Later, with inductive coding and abductive research logic we build a transferable design-and-evolution framework for PMS in public networks.

The paper contributes to scholarship and practice in two ways. Conceptually, it reframes network performance management as an iterative, governed design process rather than a one-off technical exercise, specifying the triggers (policy cycles, evaluations, IT shifts, stakeholder signals), lifecycle phases (design, use, adjustment, consolidation) and building blocks (journey–achievement–impact logic, contribution metrics, efficiency ratios, data pipeline, and governance arenas) that enable evolution. Empirically, it offers the first longitudinal account of PMS change in large trans-national public network for SME support. Practically, it distils actionable guidance for policymakers and network managers on how to measure the coordination that creates value, not only end outcomes, without sacrificing comparability across a large, heterogeneous system.

The remainder of the paper is structured as follows. Section 2 develops the theoretical framework, reviewing existing models of PMS evolution and highlighting their limitations when applied to public sector networks. Section 3 presents the methodology, outlining the longitudinal case study design, data sources, and abductive analytical strategy. Section 4 reports empirical findings, first tracing the evolution of the EEN’s PMS across successive cycles and then presenting the generic framework derived from coding and abductive reasoning. Section 5 discusses the theoretical contributions, policy and managerial implications, and avenues for future research. Finally, Section 6 concludes the paper.

## 2. Theoretical framework

Understanding how and why performance management systems (PMS) evolve in public sector networks requires grounding in the broader transformations that have taken place in public administration and governance. Over the past four decades, public sector management has shifted from traditional hierarchical models to more decentralized, interactive, and collaborative forms of governance. These changes have reshaped not only how public services are delivered but also how performance is conceptualized, measured, and managed ( Gunn *et al.*, 2021; Klijn *et al.*, 2025).

Performance measurement is generally defined as the process of assessing the efficiency and effectiveness of past actions ( Neely *et al.*, 1995). Within this context, PMS refers to a coordinated set of performance indicators or metrics used to interpret and understand the performance of an organizational entity ( Carlsson-Wall *et al.*, 2016). At their core, PMS are information systems that transform input data into actionable performance metrics, enabling managers to assess progress, provide feedback, and steer future actions ( Burney & Matherly, 2007). These systems also support the implementation of strategy, process control, communication, and the articulation of organizational priorities ( Kaplan & Norton, 2000; Wouters & Sportel, 2005).

While much research on performance systems distinguishes between *performance measurement* (the selection and definition of indicators) and performance management (the use of those indicators to steer behaviour and learning ( Bititci, 2015; Smith & Bititci, 2017) this study adopts an integrative view. The analysis treats the PMS not only as a measurement architecture but also as a managerial process through which indicators are interpreted, debated and re-designed. This broader perspective allows the study to capture how the EEN uses performance information within governance routines, evaluation practices and redesign cycles.

In the public sector, PMS are designed to enhance efficiency and effectiveness across public programmes and institutions. While primarily intended to support rational decision-making, they may also serve symbolic or ritualistic purposes ( Pollitt, 2013). PMS typically rely on decision-relevant performance indicators, often benchmarked against internal standards or external norms ( Blalock, 1999), to inform systematic reporting to administrative and political stakeholders ( Poister, 2008). The purposes served by these systems are multiple and interrelated, ranging from accountability, learning, and policy revision to planning and operational control ( Behn, 2003; Deschamps & Mattijs, 2018). Importantly, PMS data is also used for evaluation purposes, though a distinction is made between ongoing performance management and periodic evaluations that assess the broader relevance and impact of policies ( Perrin, 1998).

The evolution of public performance management cannot be understood without considering the wider paradigmatic shifts in public sector governance. The traditional public administration (TPA) model, dominant throughout much of the twentieth century, relied on formal hierarchies, standardized procedures, and rule-bound bureaucracy to ensure neutrality and accountability in service delivery ( Hughes, 2017; Osborne, 2006). However, this model became increasingly inadequate in the face of complex, multi-sectoral challenges and the growing scale of public service demands, what Rose (1981) described as the “Big Government” problem. Emerging in response, the New Public Management (NPM) paradigm introduced private-sector management techniques to the public domain. It promoted output-based accountability, market mechanisms, performance contracts, and customer orientation. Public managers were encouraged to adopt tools such as benchmarking and performance indicators, enabling them to “steer” rather than “row” ( Osborne & Gaebler, 1992). While NPM contributed significantly to the institutionalization of PMS, it also introduced fragmentation and often overemphasized measurable outcomes at the expense of broader public value or collective governance goals. To address the limitations of both TPA and NPM, a third paradigm, the "network governance", gained prominence. This approach recognizes that many policy problems are “wicked” and cannot be solved by single organizations acting in isolation. Instead, they require coordinated efforts among interdependent actors from public, private, and nonprofit sectors ( Klijn *et al.*, 2025; Koppenjan *et al.*, 2019). Governance networks are marked by horizontal coordination, shared decision-making, and mutual interdependence. In such settings, traditional hierarchical control mechanisms become less effective, and performance cannot be solely attributed to individual actors. This presents unique challenges for the design and evolution of PMS, which must now account for multiple institutional logics, diverse stakeholder interests, and distributed accountability ( Vikstedt & Vakkuri, 2025).

The literature on network governance draws from several traditions, each emphasizing different facets of networked interaction. The policy networks tradition examines the influence of interest groups on public policymaking; the service delivery tradition focuses on coordination challenges in fragmented service systems; and the managing networks tradition explores how complex policy problems are addressed through inter-organizational collaboration ( Kickert *et al.*, 1997). Despite their differences, these traditions converge around the idea that outcomes are co-produced by multiple actors, and that performance is the emergent property of interactions rather than the result of any single organization’s actions.

Supporting SMEs through public networks constitutes a complex governance challenge ( Knox & Arshed, 2022; Pulka *et al.*, 2021). Problems in this domain are rarely technical or clearly defined. Instead, they are substantively complex, marked by competing definitions of problems and diverse interpretations of success ( Klijn *et al.*, 2025). Actors in these networks like national governments, regional agencies, business support organizations, and SMEs themselves, often hold divergent views on priorities, solutions, and desirable outcomes. Moreover, wicked problems, such as internationalization, innovation diffusion, and sustainability, cut across economic, social, and regulatory domains ( Benito & Meyer, 2024). These issues are not only difficult to solve, but also difficult to measure. Performance information does not always reduce uncertainty, and can sometimes exacerbate disagreement, as actors interpret the same data in different ways ( Baekgaard & Serritzlew, 2015). In addition, public debates and political discourse can influence how performance is framed, perceived, and acted upon ( Baekgaard *et al.*, 2019).

From an organizational theory perspective, networks are understood to emerge when organizations require resources controlled by others and engage in sustained interaction to access them ( Levine & White, 1961; Rogers & Whetten, 1982). These interactions are especially relevant in the delivery of complex services and are governed by coordination mechanisms that range from formal to informal ( Park *et al.*, 2021). As such, network effectiveness depends not only on structure but also on the nature of coordination ( Hjern & Porter, 1981; Raab *et al.*, 2015). Indeed, at the heart of networked governance lies the challenge of aligning heterogeneous coordination logics. Public agencies often operate under bureaucratic logics emphasizing control, accountability, and compliance; private-sector actors tend to prioritize flexibility, competition, and innovation; and nonprofit organizations typically rely on trust, shared values, and informal norms. Herranz (2007), Herranz (2010) synthesizes these into three ideal coordination orientations: bureaucratic, entrepreneurial, and community-based. Managing these conflicting logics involves balancing consistency, adaptability, and collaboration, a dilemma he calls the “management trilemma.” Each orientation implies distinct understandings of performance and demands different design features in the PMS. Therefore, network managers must not only coordinate actions but also reconcile competing rationalities within the performance system itself.

Another critical element lies in the insufficient integration of complexity into existing PMS models. Collaborative networks—particularly those involved in SME support—operate under conditions of substantive, strategic, and institutional complexity ( Hovik & Hanssen, 2015). Substantive complexity arises from divergent perceptions among actors regarding goals, priorities, and the very definition of success. Strategic complexity reflects the shifting interests and power dynamics within and across network participants, while institutional complexity stems from varying legal, administrative, and cultural frameworks across jurisdictions. Yet, much of the existing PMS literature assumes a level of stability and uniformity that does not reflect these realities. As Klijn *et al.* (2025) argue, PMS in governance networks must contend with these multifaceted challenges, but most models have yet to adequately account for them.

In this complex environment, the design of PMS requires more than the technical selection of indicators. Logic models have emerged as useful heuristic tools for aligning strategy with measurement. A logic model articulates the hypothesized causal pathway from inputs and activities to outputs and outcomes. It facilitates stakeholder engagement, clarifies assumptions, and supports the planning, monitoring, and evaluation of public programmes ( Frechtling, 2025; Hatry, 2006; Julian, 1997; Kaplan & Garrett, 2005). Because logic models are collaboratively developed, they serve as boundary objects that bridge diverse perspectives, making them especially well-suited for public networks where agreement on objectives and success criteria must be negotiated. Herranz, Jr. (2010) adapted logic models for use in network settings emphasizing their capacity to integrate performance measurement with collaborative governance. In these contexts, logic models offer structure without rigidity and can accommodate dynamic changes in strategy or coordination arrangements. They also help address a key gap in the literature: the disconnect between network coordination processes and measurable outcomes ( Koppenjan, 2008). By foregrounding causality, shared goals, and measurable results, logic models support the design of PMS that are not only technically robust but also politically and operationally feasible in networked contexts.

While logic models provide an entry point for PMS design, they do not fully explain how PMS evolve over time. For this, scholars have proposed conceptual frameworks such as the performance management life-cycle model ( Gutierrez *et al.*, 2015; van Helden *et al.*, 2012) in which PMS development typically follows four interrelated stages: design, implementation, use, and assessment. In the design phase, objectives and indicators are defined in alignment with strategy. Implementation involves system rollout and integration into organizational routines. The use phase encompasses the application of performance data for decision-making, learning, and control. Finally, the assessment phase evaluates the effectiveness of the system and may lead to redesign. Importantly, this cycle is iterative rather than linear; organizations learn from each stage and adjust accordingly through feedback and feedforward loops.

Complementing this view, Kennerley and Neely ( 2002; 2003) and later Hald and Mouritsen (2018) propose a four-phase evolutionary model of PMS change: use, reflection, modification, and deployment. These models emphasize that PMS evolution is often triggered by internal misalignments, such as gaps between performance measures and strategic priorities, or external shocks like policy reforms or stakeholder pressure. They highlight that PMS change is not merely a technical adjustment but a social and political process, often marked by resistance, negotiation, and institutional constraints. In public sector networks, where authority and control are distributed, PMS change is particularly challenging.

Additionally, the literature from operations management and management accounting further reinforces that PMS must remain dynamic and adaptable to their environment ( Bourne *et al.*, 2000; Busco *et al.*, 2007; Cosa & Torelli, 2024; Gutierrez *et al.*, 2015). Evolution may be deliberate, as in formal redesign processes, or emergent, resulting from informal reinterpretations or the gradual drift of measurement practices over time. Scholars have highlighted the role of informal systems, organizational routines, and learning processes in shaping the effectiveness and trajectory of PMS ( Braz *et al.*, 2011; Deschamps & Mattijs, 2018; Wouters & Sportel, 2005). From an institutional perspective, PMS are socially constructed systems whose meaning and relevance are constantly negotiated across actors and contexts ( Andon *et al.*, 2007; Quattrone & Hopper, 2001; van de Ven & Poole, 1995).

These models fall short when applied to public sector networks, which are characterised by distributed governance and multiple institutional layers. In these contexts, PMS development emerges through negotiation among diverse actors with often competing priorities, making the process externally shaped, co-produced, and subject to continuous adjustment rather than linear progression. Moreover, these models underplay the role of exogenous triggers which frequently redefine measurement priorities and indicators. Unlike traditional models that frame change as a product of internal learning, PMS evolution in public networks is often reactive and shaped by external inputs ( Agostino & Arnaboldi, 2018). The process is also non-linear and iterative, involving ongoing adjustments to indicators and metrics in response to operational frictions and shifting goals. In addition, the coexistence of conflicting performance logics across governance levels and the lack of attention to infrastructural supports further complicate PMS evolution in networks ( Vikstedt & Vakkuri, 2025). These dynamics underscore the need for new conceptual tools that go beyond single-organisation assumptions to capture the complex, negotiated nature of PMS change in public sector networks.

Taken together, these theoretical perspectives provide a rich foundation for analyzing the evolution of PMS in public sector networks. The transition from hierarchical government to networked governance, the interplay of diverse coordination logics, the potential of logic models for structuring performance expectations, and the dynamic, iterative nature of PMS development point to the need for nuanced, context-sensitive approaches. This study applies these lenses to a longitudinal case of a large public network, using an abductive approach to build a framework that integrates the theoretical insights discussed above with the empirical realities of performance management in public networks.

## 3. Methodology

This research adopts a qualitative, longitudinal single-case study design to examine the evolution of the PMS within the EEN, a European network that supports SMEs. The study focuses on how performance indicators, measurement tools, governance arrangements, and managerial practices evolve over time in response to institutional pressures, policy priorities, and learning dynamics within a multi-level, trans-national network structure.

The case study approach is justified given the exploratory nature of the research question, which seeks to understand how PMSs are constructed and transformed over an extended time horizon. Following Yin (2018), the single-case design is appropriate when the case is critical, revelatory, and longitudinal, offering a rare opportunity to observe organizational phenomena as they evolve in real time within their natural context. The EEN case is uniquely suitable given its temporal depth (2008–2025), pan-European structure, public governance logic, and exposure to formalized performance regimes from the outset.

The research is grounded in an abductive research logic. Abduction allows the researcher to incorporate unexpected observations and practical reasoning into theory-building, especially suitable for institutional and processual research domains like PMS development. This epistemological orientation also supports a practice-based view of PMSs ( Nicolini *et al.*, 2016), not just as technical tools, but as evolving artefacts embedded in socio-material routines, institutional logics, and political dynamics ( Jansson *et al.*, 2020).

### Case design and units of analysis

The EEN constitutes a single, coherent European network of regional business-support organisations operating across all EU regions and associated countries under the Single Market Programme ^6^. It is co-financed by the European Commission through multi-annual grants and is mandated to help SMEs innovate, grow, and internationalise, while contributing to EU objectives on competitiveness, sustainability, digitalisation, resilience, and entrepreneurship. The network works through a client-centric logic, integrating first-level information services and second-level, tailored advisory and partnering services that are expected to generate measurable business impact for SMEs. The EEN comprises almost 600 partner organisations operating within a multi-level governance architecture that includes the European Commission/EISMEA ^7^, national and regional consortia, and cross-cutting bodies such as the Representative Body and Thematic and Sector Groups. These layers embody different roles, responsibilities and coordination functions, which are central to understanding how the PMS evolves within the network.

EEN is theoretically and empirically suited for a study of PMS evolution for three reasons. First, it is the world’s largest public network supporting SMEs, offering a rich setting to observe how PMS accommodate multi-actor coordination, EU-level steering, and local delivery. Second, the programme spans multiple cycles providing temporal depth to trace evolution. This temporal structuring allows the research to trace institutional layering and shifts in performance logic over time. Third, the lead researcher’s extended professional experience within the network afforded access to archives and meetings that are typically difficult to observe, while also necessitating explicit reflexive strategies to mitigate role-related bias. Indeed, to address potential bias associated with this position, we employed (i) source triangulation (archives, administrative data, observations), (ii) a structured audit trail linking claims to documents and dated events, and (iii) reflexive memoing to separate descriptive notes from interpretive commentary. No personal or commercially sensitive information is reported; evidence is presented in aggregate or anonymised form consistent with organisational guidelines.

The empirical focus is the EEN ‘s PMS, with data collected from the CESEAND consortium ^8^ (the Andalusian node of EEN) and its engagement with EU-level structures.

### Data collection

The research uses multiple data sources in line with case study best practices. Data were collected over several years through document analysis, direct observation, and memo writing, enabling both retrospective and real-time insights into the evolving PMS.

Primary data consisted of compilation of archival documentation as well as researcher-generated memos from direct observations. Memos were produced systematically during participation in EEN governance arenas such as Steering Committee meetings, Board sessions, KPI Working Groups, and thematic training events between 2015 and 2023. Observations were conducted during both formal meetings and informal exchanges, enabling the researcher to trace sensemaking processes and disagreements around indicator definitions, evidence rules, and KPI interpretation. These materials constitute the core ethnographic-style evidence used to identify recurring mechanisms and interpretive patterns in the PMS evolution.

The researcher-generated memos constitute rich ethnographic material capturing the deliberations, rationales, disagreements and sensemaking expressed by key actors involved in PMS design and implementation. Because these governance arenas (e.g., KPI Working Group, Steering Committees, Technical Boards) are precisely where PMS definitions and revisions are debated, they provide direct access to the motives behind PMS decisions. Although complementary interviews could have added additional perspectives, the density of these deliberative settings provides sufficient depth for the aims of this study.

The empirical material is classified into four main categories, as summarized in Table 1 below:

**Table 1.** Underlying data sources used in the study.

Category	Document Title	Code
**EU Call for** ** proposals**	Call for proposals CELEX_C2006_306_07_EN	1.1
	Terms of reference EEN 2017–2018_en	1.2
	Call for proposals_smp-cosme-2021-een_en	1.3
	Call for proposals_smp-cosme-2024-een_en	1.4
**Evaluation** **reports**	Evaluation report_eip_en 2011	2.1
	Evaluation report_eaci_2011_en	2.2
	Evaluation report_EEN_2008–2014	2.3
**Memos**	Memo PMS used by the EEN period 2008–2025	3.1
	Memo CESEAND Plus Period 2015–2016	3.2
	Memo CESEAND Plus Period 2019–21	3.3
	Memo Working Group on Measurement and KPIs	3.4
	Memo Training Improve Your Achievements	3.5
	Memo Thematic Group Start-up and Scale-up	3.6
**Complementary** ** sources**	Conference proceedings on EEN (2008–2020)	4.1
	Report EEN Our Story 2008–2020	4.2

The calls for proposals provide insight into the formal expectations and design logic underpinning each programming cycle. The external evaluation reports, authored by independent contractors on behalf of the European Commission, offer a meta-assessment of the EEN’s performance effectiveness, relevance, and coherence with policy objectives. The researcher memos (developed between 2015 and 2023) are based on participation in internal meetings, working groups, training sessions, and archival analysis, including grants agreements and project reporting. These constitute primary ethnographic style data, capturing practices, conversations, concerns, and interpretations among EEN members. Finally, the complementary sources help contextualize the Network’s institutional narrative and learning trajectory. All these documents are included as underlying data ( Ruiz-Coupeau, 2025), ensuring transparency and traceability of interpretations.

### Data analysis

The data analysis proceeded in two distinct but complementary stages.

The first stage aimed at describing the evolution of the PMS across different phases. To achieve this, we employed temporal bracketing ( Langley, 1999), a processual analysis technique that structures longitudinal data into successive periods separated by identifiable shifts. Temporal bracketing allows the researcher to capture both continuity and change by segmenting a process into analytically meaningful intervals, while preserving the temporal ordering of events. This method is particularly suitable for studying evolving organizational systems, as it highlights how earlier configurations create the conditions and constraints for subsequent developments.

In practice, we reconstructed a timeline of the EEN’s PMS by bracketing the material into programming phases (2008–2012; 2012–2015; 2015–2020; 2021–2025; and a forward-looking phase beyond 2025). Within each bracket, we organized archival documents and observation notes to identify key design decisions, governance arrangements, data infrastructure shifts, and revisions to indicators/KPIs and quality processes. This structuring yielded a longitudinal narrative of PMS evolution that made visible the episodic, cyclical character of change, while also clarifying how triggers and building blocks carried over across successive funding periods.

The second stage moved beyond description to develop a conceptual framework explaining the observed evolution. Here, the coding procedure combined systematic qualitative coding with an abductive logic of inquiry. Following abductive reasoning ( Peirce, 1934; Timmermans & Tavory, 2012), the study moved iteratively between data and theory to explain surprising patterns observed during the evolution of the performance management system. This approach was particularly suited to our aim of reconstructing the evolution of the EEN’s PMS and developing a more generalizable framework for PMS design and evolution in public sector networks ( Pfister *et al.*, 2023).

The coding and analysis were conducted by the first author, who was directly involved in the archival and observational data collection. Coding results and category definitions were periodically reviewed and discussed with co-authors.

The first stage of analysis involved *open coding*, where each document was read iteratively, and relevant passages were coded using descriptive terms. Codes captured three broad domains: performance artefacts (e.g. indicators, dashboards, reporting formats, databases); practices and routines (e.g. peer learning, mentoring, knowledge sharing, training); and governance mechanisms (e.g. working groups, steering committees, evaluation protocols). This process generated a comprehensive inventory of elements that constituted the PMS.

In the second stage, *axial coding* was used to cluster related codes into higher-order categories that expressed the mechanisms of PMS evolution. For instance, “EU calls,” “grant agreements,” and “policy cycles” were grouped under the category of policy and funding triggers. Likewise, “external audits,” “evaluation recommendations,” and “impact indicators” were consolidated into a category representing evaluations as redesign triggers. Finally, “client journey,” “achievements,” and “impact pathways” were grouped into a category of measurement logic. Axial coding thus enabled the identification of recurrent dynamics linking codes across sources and over time.

The final stage, *selective coding*, connected these axial categories into a coherent explanatory model. Here, abductive reasoning was central: we sought the most plausible structure to integrate empirical findings with theoretical insights on performance systems, organizational learning, and network governance. This process led to the construction of a generic framework for the design and evolution of PMS in public networks, which integrates three interconnected layers.

Through this abductive process, the framework was not “discovered” inductively as given, but constructed as the most plausible explanatory account of PMS evolution. It is grounded in the detailed coding of EEN documents and observations, yet abstracted to a level that renders it transferable to other public sector networks. Thus, abduction provided a bridge between empirical richness and theoretical generalisation, strengthening both the explanatory power and practical relevance of the framework.

## 4. Findings

These findings are presented in two steps: (1) the evolution of PMS across the different phases, and (2) the construction of a generic framework for the design and evolution of PMS in public networks:

### 4.1. Evolution of PMS in 15 years

The findings are structured around distinct phases in the evolution of the EEN (2008–2012; 2012–2015; 2015–2020; 2021–2025; and a forward-looking phase beyond 2025). These phases were identified by the researcher based on shifts in PMS structures, with each period characterized by relative stability before subsequent changes emerged. The analysis indicates that the PMS did not develop along a linear trajectory of technical refinement, but rather evolved through cyclical patterns.

Across five phases, the EEN’s PMS shifted from an activity/output-oriented dashboard to a networked, client-journey architecture that tracks achievements and impact. The early list of indicators suite primarily counted events, contacts and hours; subsequent cycles introduced logic-model elements and, later, a formal client-journey structure linking advanced advisory and partnering services to measurable achievements and business impact for SMEs. In the most recent phase, a consolidated set of key performance indicators (KPIs) and efficiency ratios embeds network logics (e.g., contributions across consortia) and emphasises coordination, capacity building and quality management.

***Phase 1 (2008–2012): activity and output orientation.*** In the first phase, services were organised under three broad objectives:information/feedback and internationalisation, innovation and technology/knowledge transfer, and support to participate in Research and Technological Development (RTD) projects —consistent with the Competitiveness and Innovation Programme (CIP) ^9^. Monitoring focused on activity volumes and immediate outputs. For instance, EEN members reported counts of events, media outputs (press articles, newsletters), brokerage activity, and time allocations, with limited capture of downstream effects. These indicators demonstrate a strong emphasis on promotion, service reach and transactional brokerage metrics in the early PMS design.

The external evaluation covering the period highlighted high client satisfaction and European added value, while calling for clearer vision/leadership, stronger visibility, and “more impact-oriented monitoring”. It also noted the introduction of a new IT system whose integrative capacity had yet to be assessed and observed that policy feedback functions required substantial effort with uncertain regulatory impact, points that foreshadowed later PMS redesigns.

**Figure 1.**
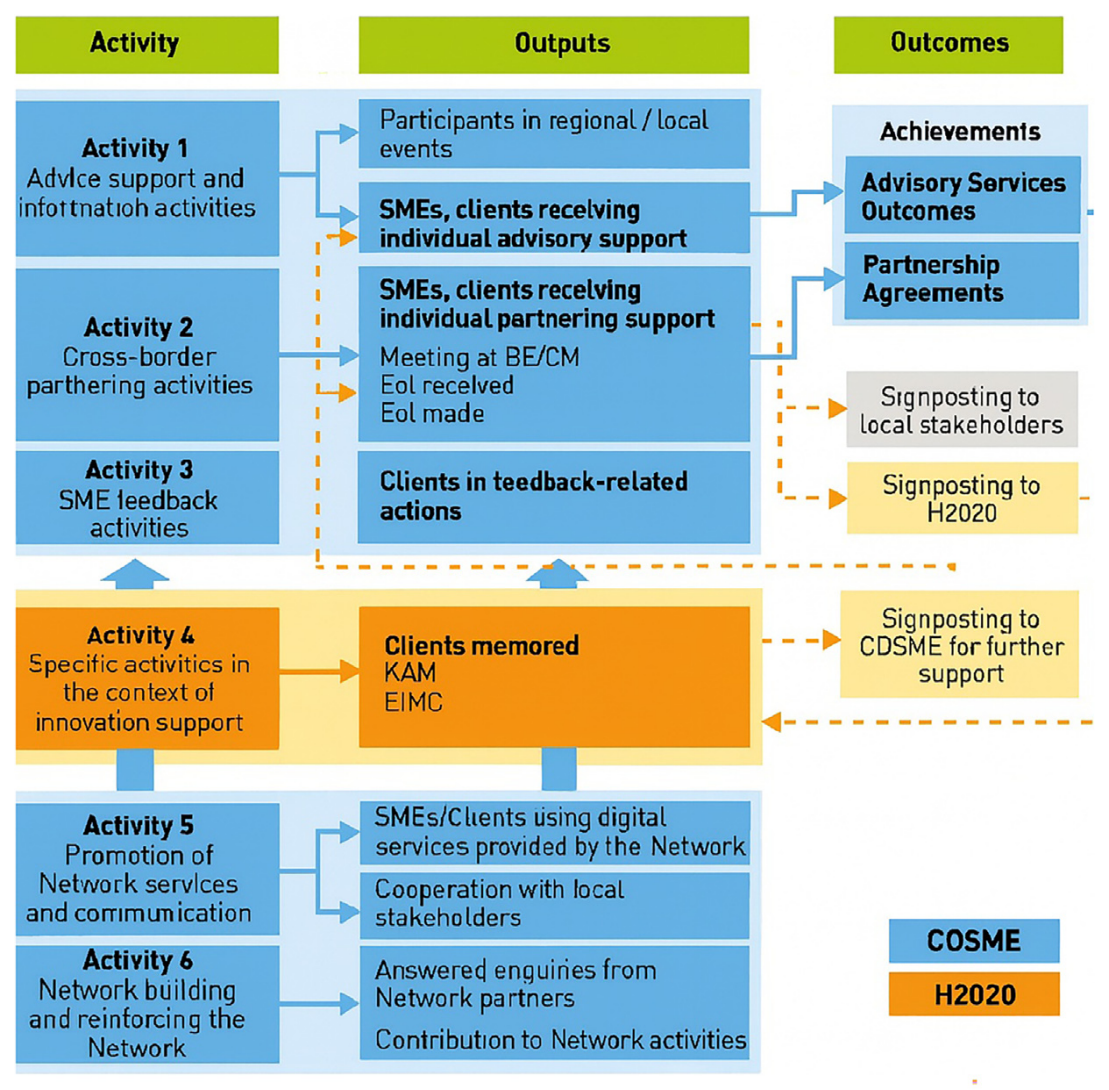
EEN activities, outputs and outcomes. Retrieved from document “1.2 Terms of reference EEN 2017-2018_en” Ruiz-Coupeau (2025).

***Phase 2 (2012–2015): consolidation and preparation for redesign.*** The second phase consolidated service families and deepened innovation and partnering activities, but the monitoring system remained largely outcome-centric while groundwork was laid for a more explicit logic-model approach as can be seen in Figure 1 where activities, output and outcomes are graphically displayed.

Three of the six standard activities of the Network can be understood through a logic model approach, where each activity is designed to generate specific outputs that ultimately lead to outcomes (SME achievements). Advisory, support, and information services (activity 1) provide SMEs with direct guidance on EU policies and programs, producing outputs such as informed businesses and tailored advice, which in turn strengthen SMEs’ competitiveness and innovation capacity (Advisory service outcomes). Cross-border cooperation activities (activity 2) generate outputs like business meetings, which translate into outcomes such partnerships, technology transfer agreements, and research collaborations (Partnership agreements). Similarly, innovation support activities (activity 4) deliver outputs such as diagnostic reports and action plans for SMEs, along with coaching connections through the Key Account Manager system; the outcome is stronger innovation management and higher success in European R&D initiatives (Advisory service outcomes).

Besides, network governance arrangements (e.g., EU-level coordination with a representative body) began to formalise communication and change-management channels, setting the stage for more coordinated PMS development across countries and consortia.

***Phase 3 (2015–2020): introduction of the client-journey.*** A major step-change occurred with the explicit adoption of a client-journey logic that sequences first-level (basic) and second-level (advanced) services. Second-level services, either advisory or partnering, were now expected to culminate in Achievements (milestones) and, ultimately, business impact for SMEs (e.g., increase in market share, turnover, cost optimisation/savings, jobs, quality and innovation outcomes). This recast the PMS from counting isolated interactions to tracing cumulative value over time, aligning service delivery with measurable milestones and impact categories.

Concurrently, network learning infrastructures matured. Sector and Thematic Groups expanded their role as competence centres, supporting advanced casework and disseminating methods and tools across consortia—an enabling condition for consistent measurement and comparable “achievement” definitions.

**Figure 2.**
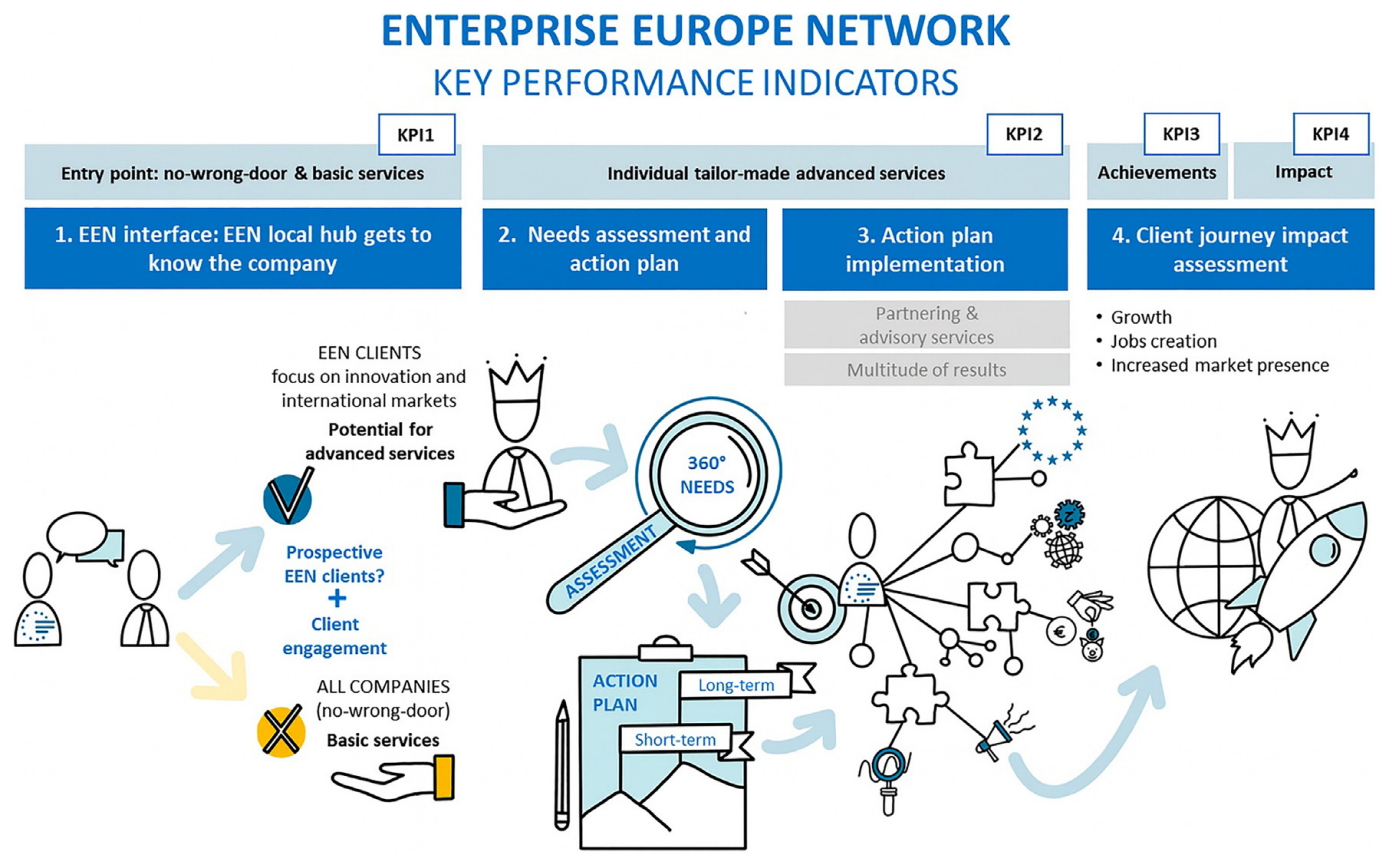
Client journey and corresponding KPIs. Source ( European commission, 2021).

***Phase 4 (2021–2025): KPI consolidation, efficiency ratios and network logics.*** Under the Single Market Programme, activities were streamlined into four pillars: value-added services to clients, promotion/communication, network development & capacity building, and coordination & quality management. The PMS is consolidated integrating KPI suite (KPI1–KPI4) focused in the client journey as shown in Figure 2:

Four efficiency ratios completed the client-journey architecture and embedded network coordination. These ratios track efficiency and effectiveness (e.g., achievements per client, clients reporting impact per unique client in the client journey, achievements per FTE, average impact per client), sharpening attention to value creation rather than volumes alone. New metrics include unique clients in the client journey (KPI2) and impact on clients (KPI4). Activity indicators capturing contribution metrics are included: contribution to other partners’ journeys and network development activities. SME feedback is also included as an activity indicator.

Governance mechanisms were strengthened to support this shift. A representative body (plenary and bureau) acts as a sounding board, relays network views to the Commission and Agency, and leads the implementation of changes across countries; Sector and Thematic Groups function as hubs for expert exchange, common approaches and learning, including feedback on Single Market obstacles or disruptions. Importantly, temporary working groups were convened with a limited mandate to redesign the PMS and KPIs, evidencing a deliberate, participatory change process.

***Phase 5 (2025 →): stabilising the model and extending project management.*** The next cycle retains the four activity pillars and explicitly adds project management as a fifth activity for all consortia, signalling institutionalisation of coordination and quality routines alongside service delivery. The continuity of the KPI suite and quality ratios indicates consolidation of the client-journey measurement model, with an emphasis on cross-consortia contributions and capacity building as levers of network-level performance.

Taken together, the EEN case shows a managed yet non-linear evolution of PMS: external accountability demands, and internal learning pressures periodically align to produce redesigns; governance forums translate these pressures into shared definitions and routines; and the measurement focus progressively shifts from outputs to journey-anchored achievements and impact, with explicit metrics for networked production and quality. The result is a PMS that better fits the realities of collaborative SME support while retaining comparability and steerability across a large, heterogeneous transnational network.

### 4.2. Construction of a generic framework for the evolution of PMS in public networks

The generic framework constructed through an abductive process, weaving together archival evidence (calls for proposals, grant agreements, evaluations, Commission communications) with direct observations of the EEN at both European and consortium level. This section identifies the mechanisms that explain why the PMS transitions from one phase to another, complementing the descriptive temporal analysis in Section 4.1. These mechanisms (policy and funding cycles, evaluation pressures, shifts in IT infrastructure and stakeholder signals) clarify why redesign occurs. Abduction was crucial because neither a purely deductive application of theory nor an inductive generalisation from observations could fully account for the distinctive dynamics of PMS in large, contractual public networks. Instead, by iteratively moving between theory and data, we inferred a model that explains both the rigidity of KPIs within funding cycles and the recurrent, structured evolution of the PMS across cycles.

The coding of documents and memos revealed recurrent patterns that were grouped into higher-order elements. These ranged from external forces, such as policy cycles or evaluation pressures, to internal processes of PMS development, and to the technical and institutional components that stabilise measurement practices.

Table 2 presents the analytical coding scheme that underpins the development of the performance management system (PMS) evolution framework. The table integrates empirical evidence and analytical interpretation, showing how specific data excerpts informed the identification of broader conceptual categories. The first column lists the open coding categories derived from the primary and secondary sources. These are followed by the corresponding data references, empirical grounding, and their clustering into axial and selective coding dimensions. This structure clarifies the analytical path from first-order evidence to the conceptual elements of the PMS framework, highlighting how contextual triggers interact with lifecycle phases and contribute to the emergence of stable building blocks within the system.

**Table 2.** Empirical foundations and coding progression from open codes to axial and selective categories.

Open coding categories	Supporting sources	Empirical grounding	Framework element (axial categories)	Framework layer (selective codes)
Multiannual programmes, calls, grant agreements	1.1; 1.3; 1.4; 3.1	Multiannual programmes and their call texts form the backbone of the PMS, determining when redesign cycles occur and what strategic, operational, and reporting expectations must be adopted. Calls consistently define mandatory work packages, KPIs, service components and evidence protocols. For example, call 1.4 states: *“The common starting date for all Enterprise Europe Network projects is 1 July 2025… project activities must be organised in the following mandatory work packages.”* This illustrates the contractual rigidity and harmonised timing that condition when PMS revisions can take effect. Earlier calls show similar structuring: the 2006 call established the Network’s initial architecture, while the 2021 call reframed the Network as *“a client-centred service ecosystem,”* anticipating a shift from activity-based to journey- and impact-oriented measurement. Memo 3.1 confirms this direct linkage, noting that PMS changes *“followed programme-cycle transitions and were shaped by revised call requirements,”* with shifts between CIP, COSME and SMP corresponding to successive reconceptualisations of achievements, contributions and impacts. These sources collectively show that multiannual programmes act as structural triggers for PMS redesign. The PMS evolves in synchrony with programme cycles: each new cycle sets conditions for a new PMS version, requiring reinterpretation of existing indicators and prompting adoption of updated measurement architectures.	Policy & funding cycles	Contextual triggers
CRM/reporting portals, templates, evidence protocols	1.2; 1.3; 3.1; 3.2	The CRM, along with its templates, evidence protocols and metadata rules, forms the key infrastructure through which the PMS is enacted and stabilised. The ToR 1.2 highlights the centrality of CRM fields and mandatory evidence rules, noting: *“Indicating which Network service led to the Achievement is mandatory,”* and requiring all achievements to use standardised database fields. Thus, the CRM acts not only as a repository but as a structuring device that defines what can be counted, how it must be described, and what qualifies as evidence. The 2021 Call (1.3) reinforces this role by embedding CRM use into contractual obligations: achievements, contributions and impacts must follow predefined metadata structures, institutionalising the CRM as the PMS’s measurement backbone. Memo 3.1 confirms that PMS changes “ *followed the adjustments made in the Achievements Database*,” showing that database logic both constrains and enables indicator evolution. Memo 3.2 provides insight into how partners experience these constraints in practice, describing recurring discussions on consistency, evidence quality and the challenge of aligning interpretations across consortia. Integration efforts were needed precisely because CRM rules generated different understandings of documentation requirements. Together, these sources show that CRM and evidence protocols function as infrastructural triggers of PMS evolution, shaping partner behaviour and interpretive practices.	IT & reporting infrastructures	Contextual triggers
External evaluations, recommendations	2.1; 2.2; 2.3; 3.2; 3.4	External evaluations have been decisive legitimising triggers for PMS reform, highlighting shortcomings and creating pressure for redesign. The 2011 EACI Evaluation notes that the Agency helped partners *“become aware of the importance of monitoring performance”* (2.2) while also calling for *“a more flexible and supportive approach”* to monitoring, combining validation with critique and prompting reconsideration of the existing measurement logic. The 2008–2014 Final Impact Evaluation likewise provides strong empirical evidence that EEN-supported SMEs outperform non-clients, reporting that *“Network clients achieve higher average turnover and employment growth compared with non-clients”* (2.3). Yet this positive effect exposes a PMS weakness: the system was not capturing such impacts reliably, implicitly questioning indicator adequacy and calling for stronger outcome measures. Internal documents show how these critiques reverberated through the Network. Memo 3.4 records partners’ reactions, stating: *“Current achievement reporting failed to adequately capture the real value provided to SMEs.”* Memo 3.2 similarly demonstrates how evaluation insights became routine topics in governance discussions. Taken together, external evaluations act not merely as assessments but as structural pressures, shaping policy expectations and influencing internal sensemaking about how the PMS must evolve.	External evaluations	Contextual triggers
SME surveys, partner feedback, EC/EISMEA guidance	1.3; 2.3; 3.2;3.3; 3.4	SME survey results from 2.3 showed that *“Network clients achieve higher average turnover and employment growth compared with non-clients”*. This reinforced the need to strengthen the PMS’s capacity to capture and evidence impact. Meanwhile, EC/EISMEA guidance in the Calls (3.2 and 3.3) emphasises a service ecosystem that is *“client-centred and impact-driven”,* signalling upward expectations to shift PMS logic from activity recording to value creation. Partner feedback in memos expresses frustration with the limitations of existing indicators. Memo 3.2 notes that reporting inconsistencies required governance intervention to *“support integration and alignment of reporting practices.”* Memo 3.4 similarly documents partners’ concerns that current measures *“failed to adequately capture the real value provided to SMEs.”* Together, these sources show that SME surveys, partner experience, and EC guidance form a convergent set of stakeholder signals. They highlight two main pressures: (1) the need to capture deeper impact beyond outputs, and (2) the need for coherent, comparable evidence across consortia. As a combined force, these signals prompt rethinking of the PMS architecture and strengthen the case for the later adoption of the client-journey model, impact KPI logic, and efficiency ratios.	Stakeholder feedback	Contextual triggers
Call texts and grant negotiation of KPI set	1.3; 1.4; 3.4	Call texts are the formal legal and contractual locus where the PMS is defined, negotiated and codified. They specify the KPI set, indicator definitions, evidence obligations, contribution rules, metadata requirements and impact expectations, effectively establishing the PMS architecture for each multiannual period. The 2024 call (1.4) makes this explicit: *“Achievements are milestones for the clients counted under KPI2… KPI3a Advisory Achievements… KPI3b Partnering Achievements… KPI4 Impact on clients.”* This confirms that call texts are binding specifications of what is measured, how it must be interpreted and which forms of evidence are acceptable. Memo 3.4 provides insight into the negotiation phase preceding call publication, noting: *“The WG proposed revised definitions where achievements were milestones within the client journey.”* This shows that call texts finalise a longer chain of partner debates and synthesis. Memo 3.1 similarly explains that PMS definitions *“followed interpretive refinements debated within the measurement working groups before being incorporated into contractual updates,”* highlighting the iterative nature of this process. Taken together, these sources show that call texts are the formal crystallisation of the PMS redesign cycle. They embed negotiated meanings of achievements, contributions and impact into an enforceable contractual framework that governs reporting until the next programme cycle, marking the design moment where conceptual proposals become binding requirements.	Design	PMS lifecycle
Training, thematic groups, mentoring, QA routines	3.5; 3.2; 1.2	Training sessions, thematic groups, mentoring mechanisms and QA routines form the key interpretive infrastructure through which partners learn to enact the PMS and align reporting practices. Memo 3.5 shows this clearly, noting that sessions were *“interactive and grounded in live profile reviews, encouraging participants to reflect critically on their output”*, demonstrating that training functions as collective sensemaking rather than simple instruction. Memo 3.2 further illustrates how Steering Committees, Technical Boards and Working Groups act as interpretive devices that *“support integration and alignment of reporting practices”*, creating feedback loops where advisors raise challenges, clarify evidence rules, and negotiate shared conventions. The ToR (1.2) institutionalise these mechanisms by embedding training, peer exchanges and thematic groups into governance as tools for *“consistent application of Network service standards.”* They also specify QA routines for reviewing achievement profiles and verifying evidence, underscoring the need for continual interpretive calibration. Together, these sources show that training, mentoring and QA constitute the practical layer where the PMS is operationalised, interpreted and normalised. This is where partners learn how definitions apply in real cases, how evidence should be formulated, and how interpretive ambiguity is reduced forming interpretive communities that stabilise the PMS across the Network.	Appropriation	PMS lifecycle
Mid/final evaluations, review meetings, WG debates	3.2; 3.4; 2.1;2.2; 2.3	Internal reviews, mid-term evaluations and WG debates are key interpretive arenas where partners assess the PMS’s functioning and limitations. Memo 3.4 highlights evaluative tensions, noting: *“A recurring observation was that current achievement reporting failed to adequately capture the real value provided to SMEs.”* This reveals a mismatch between mandated reporting and partners’ lived experience, with WG discussions frequently problematising conceptual gaps such as the inability of earlier PMS versions to reflect client progression or multi-partner contributions. Memo 3.2 shows that such evaluation occurs not only in WGs but also in consortium governance routines, where technical boards and steering committees analyse evidence quality, interpretive inconsistencies and CRM challenges, noting that meeting structures *“support integration and alignment of reporting practices.”* These internally driven reflections are crucial for diagnosing operational problems, clarifying definitions and preparing adjustments before formal WG synthesis. External evaluations reinforce and legitimise this internal critique. The EACI evaluation (2.2) highlights gaps in impact orientation and challenges in assessing distributed value creation, prompting calls for stronger outcome measurement. Taken together, these sources show that evaluation is a multilayered and central PMS activity surfacing deficiencies, exposing conceptual misalignments and creating shared recognition of the need for redesign. This reflexive work prepares the ground for synthesis and renewal in the PMS lifecycle.	Evaluation	PMS lifecycle
WG synthesis, drafting of new indicators for next call	3.4; 3.1; 1.3; 1.4	The WG synthesis phase is the pivotal moment where evaluative concerns are translated into concrete redesign proposals for the next programme cycle. Memo 3.4 shows partners collaboratively drafting revised definitions to address weaknesses in the PMS, noting: *“The WG proposed revised definitions where achievements were milestones within the client journey and could include contributions from multiple partners.”* This reflects two major conceptual shifts: treating achievements as staged progress and recognising distributed value creation. Memo 3.1 confirms that these proposals became the conceptual basis for later PMS versions, explaining that indicator evolution *“followed the need to represent SME support more accurately by embedding milestones and contributions.”* WG synthesis therefore operates as a core generative mechanism rather than a purely advisory step. The progression from proposals to formalisation is evident in the calls. The 2021 Call (1.3) begins embedding journey-based logic, while the 2024 Call (1.4) codifies WG proposals explicitly: *“Achievements are milestones in the client journey… KPI3a and KPI3b reflect advisory and partnering achievements… KPI4 measures client impact.”* This demonstrates a direct continuity from WG deliberation to contractual codification. Overall, WG synthesis serves as the essential bridge between contextual triggers, evaluative debates and the redesign of PMS architecture. It transforms distributed experience and reflexive critique into the formal indicator frameworks that govern the next multiannual period.	Renewal	PMS lifecycle
Client-journey logic (journey → achievements → impact)	3.1;1.3; 1.4	The shift toward a client-journey logic represents one of the most substantial conceptual evolutions within the EEN’s PMS. Memo 3.1 explicitly documents the introduction of this model, stating: *“The logic model introduced the Network Client Journey, organised around seven structured activity lines with 13 KPIs and 7 activity indicators.”*. This reorientation marks a transition away from counting atomised activities toward understanding support as a progressive sequence of milestones that reflect deepening engagement and value creation for SMEs. The 2024 Call (1.4) formalises this journey logic into the contractual foundations of the PMS, specifying that *“Achievements are milestones in the client journey”*. This statement codifies the journey structure as the interpretive backbone of the entire indicator system, linking activities logically to achievements and achievements to impact. Earlier, the 2021 Call (1.3) introduced the conceptual precursor by emphasising a client-centred approach and embedding service lines that naturally aligned with a staged progression model. Together, these sources illustrate a clear developmental trajectory. This reorientation fundamentally changes what is considered meaningful performance. Instead of reporting discrete outputs, the PMS now reflects the depth, progression, and cumulative value of support, linking micro-interactions to broader SME impact pathways.	Measurement logic	Building blocks
Cross-partner assists, shared achievements	1.3; 1.4; 3.2; 3.4	A major conceptual shift in the PMS emerges when achievement reporting moves from single-partner attribution toward recognising distributed value creation across the Network. 2024 call (1.4) explicitly codifies this principle: *“Achievements can be reached with the participation of one or more Network partners (in this case they can be reported by both).”*. This indicates a formal acceptance of collaborative contributions and provides a contractual basis for multi-partner achievement reporting. Working-group discussions document the *bottom-up origins* of this shift. Memo 3.4 records partners’ debates on contribution measurement, highlighting concerns that existing definitions made collaborative work invisible. The memo states: *“The WG proposed revised definitions where achievements were milestones within the client journey and could include contributions from multiple partners.”*. This demonstrates that the recognition of cross-partner assists originated in collaborative reflection on how value is jointly created and how the PMS failed to capture it. Cross-partner assists emerged not only as an operational practice but as a new attribution logic embedded in the redesigned PMS *.*	Contribution metrics	Building blocks
Ratios (achievements per client, FTE, impact share)	1.4; 3.1	A significant development in the PMS is the introduction of explicit performance ratios, which reframe evaluation from simple output aggregation to normalised measures of efficiency and quality. 2024 Call (1.4) introduces *Efficiency Ratios*, specifying: *“R1: Achievements per unique client… R3: Achievements per full-time equivalent (FTE). Outputs are not only counted but contextualised through resource use and client impact.”* These ratios define explicit normative expectations and enable comparisons across consortia by adjusting for differences in client volumes, staff resources, or outcome distributions. Rather than simply reporting the number of achievements, partners must now meet proportional expectations, creating a performance benchmark culture within the Network. The Memo 3.1 confirms the strategic rationale behind these ratios, noting that more sophisticated metrics were needed during the transition to the client-journey model to capture “ *quality and depth of support rather than simply volume*”. These sources show that ratios emerged from the convergence of EC expectations, internal debates on quality and comparability, and the need to contextualise achievements within resource use. This shift marks a deepening of performance management logic: measurement becomes not only descriptive but evaluative, embedding performance norms and enabling benchmarking across diverse network partners.	Efficiency lens	Building blocks
Definitions, evidence rules, metadata, CRM	1.2; 1.3; 3.1	The ToR 2017–2018 (1.2) establish strict evidence rules and data-entry conventions that shape reporting practices across the Network. For example, they mandate: *“indicating which Network service led to the Achievement is mandatory”*, thereby introducing a compulsory metadata grammar that constrains how advisors document their work. Similarly, 2021 call (1.3) specifies the definitions, fields and mandatory attributes required for achievements, contributions, and impacts, reinforcing that reporting is not discretionary but guided by a shared evidence protocol embedded in the CRM. Memo 3.1 emphasises how successive PMS versions depended on changes in the Achievements Database, explaining that indicator definitions “followed the adjustments made in the Achievements Database,” demonstrating that data architecture and metadata structures actively shape what the PMS becomes. Across these sources, a clear picture emerges: definitions, evidence rules, metadata requirements and CRM structures do not merely support reporting: they constitute the *measurement grammar* that stabilises the PMS by defining what counts as an achievement, how it must be evidenced, how it is attributed, and how it is validated. This grammar provides the structural coherence necessary for network-wide comparability and reduces interpretive ambiguity, but also generates tensions that partners must negotiate through training and governance arenas.	Data pipeline	Building blocks
Representative body, thematic groups, KPI design WGs	1.2; 1.3; 3.2; 3.4	Governance structures that institutionalise consultation, feedback and decision-making are described in the calls. 2011 call (1.3) explains that the Steering and Advisory Group *“provides channels for partners to advise the Commission and the Agency”* and emphasises that annual conferences, training structures, and thematic groups support consistent Network operation.The 2017–18 ToR (1.2) further elaborate mechanisms for coordination and internal alignment across consortia. Field evidence from Memo 3.2 shows these governance devices in action, noting that Network integration was *“explicitly framed as a quality enabler, facilitated through a tiered meeting schedule (Steering Committee, Technical Board, Working Groups)”*. This confirms that the governance architecture does not merely exist on paper but actively shapes partners’ routines, interpretive processes, and alignment strategies. Memo 3.4 demonstrates how governance arenas become the sites where measurement definitions are negotiated: *“The WG proposed revised definitions where achievements were milestones within the client journey and could include contributions from multiple partners.”* The memo also captures dissent, problematisation, and the negotiation of meanings surrounding achievements, contributions, and impact. Together, these sources show that governance arenas are not passive structures but active sites of *collective design*, *interpretive coordination*, and *institutional negotiation* of the PMS.	Governance arenas	Building blocks

***Abductive construction of the framework.*** The movement from the coded categories in Table 2 to the layered framework is made possible by abductive reasoning. Abduction differs from deduction and induction: it is not a linear application of theory to data, nor a simple generalisation from observations, but a creative process of inference to the “best possible explanation” of observed patterns. In our case, archival material and direct observation produced numerous categories: policy cycles, external evaluations, IT infrastructures, stakeholder feedback, grant specifications, internal trainings, governance bodies, measurement logics, efficiency ratios, and more. At first glance, these categories seemed heterogeneous and unconnected.

Through abductive analysis, we asked what kind of organizing logic could plausibly integrate our observations into a coherent model of PMS evolution. Iterative coding and comparison revealed two striking empirical regularities. First, performance indicators were never modified within a funding period but only at its contractual boundaries, which challenges models that assume continuous adjustment of measures. Second, despite this formal fixity, the network was far from static: actors were constantly appropriating the system, reflecting on its adequacy, and preparing proposals for future redesign. These patterns could not be fully explained by conventional PMS models that emphasize incremental change and ongoing recalibration of indicators. Abduction therefore required us to reframe the cycle not as continuous evolution but as a sequence of phases (design, appropriation, evaluation, and renewal) punctuated by external contractual boundaries.

A second abductive move concerned the clustering of categories. Categories such as policy cycles, evaluations, IT upgrades, and stakeholder feedback clearly referred to forces external to the day-to-day operation of the PMS. Interpreting them as contextual triggers allowed us to explain why system redesign happens episodically. Categories such as KPI specification, trainings, evaluations, and renewal debates described the temporal unfolding of the PMS; reinterpreting them as a lifecycle of phases gave coherence to the EEN’s recurrent rhythm. Finally, categories such as measurement logic, contribution metrics, efficiency ratios, data pipeline, and governance arenas cut across cycles and triggers; abductively, it made sense to treat them as building blocks, the stable elements that make the system function at any point in time.

Thus, the abductive process did not merely sort categories but actively searched for the most plausible explanatory configuration. The result is a three-layer generic framework: contextual triggers as the outer drivers of change, the PMS lifecycle as the patterned rhythm of design and use, and the building blocks as the core architecture. Each layer gains meaning only in relation to the others: without triggers, there would be no renewal; without building blocks, appropriation would not be possible; without the lifecycle, triggers and blocks would remain unconnected. The framework therefore represents not just a classification but an abductively reasoned explanation of how PMS in public networks evolve over time.

**Figure 3.**
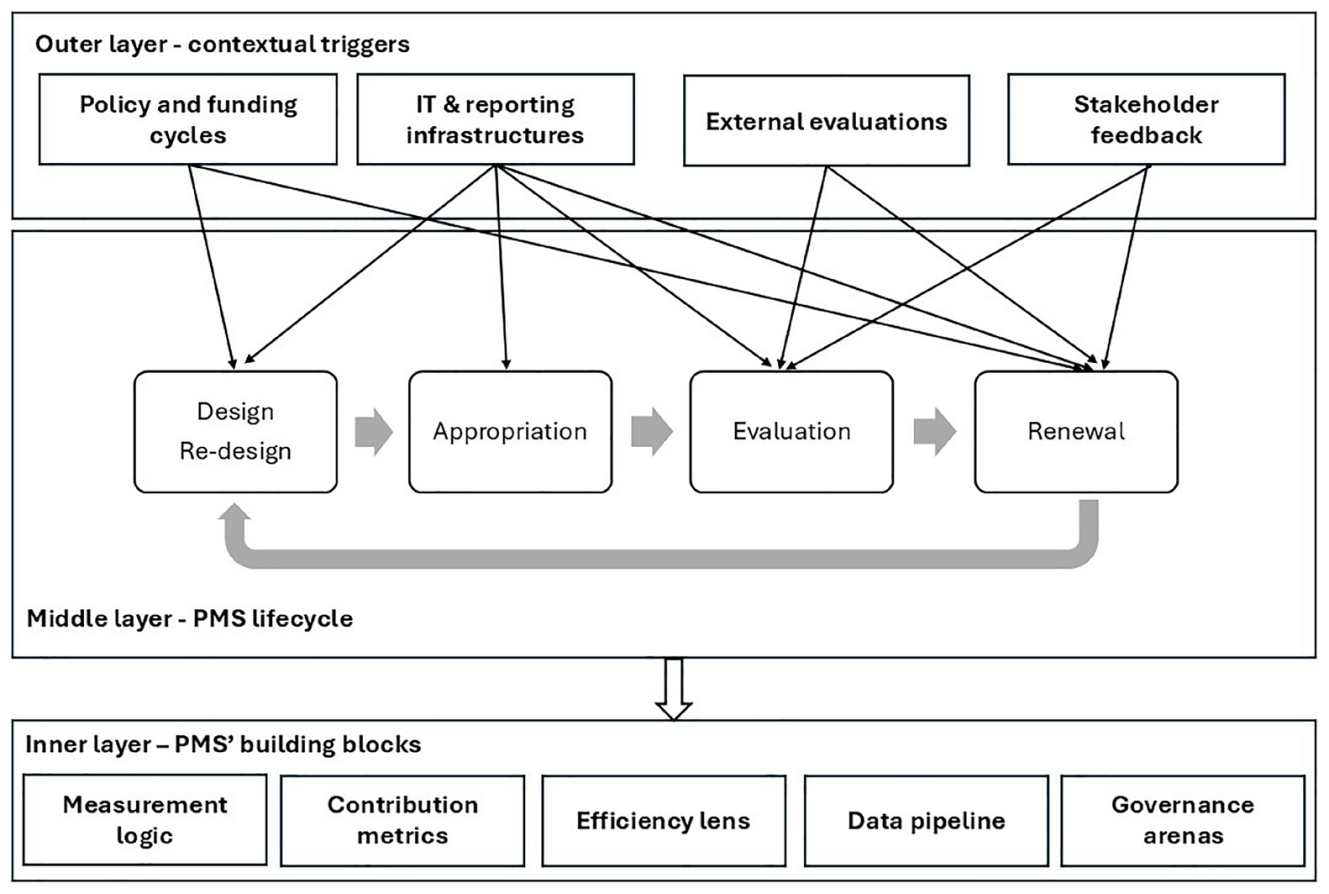
Generic framework for the evolution of PMS in public networks.

The abductive moves described above provided the interpretive logic that transformed the coded categories of Table 2 into the three-layered framework represented in Figure 3. Arrows indicate causal and iterative relationships between layers, illustrating how contextual pressures drive lifecycle processes that, in turn, produce and reshape the system’s building blocks.

This framework provides a transferable model for understanding PMS evolution in any type of public sector network:

***Outer layer: contextual tiggers.*** At the outermost level, PMS evolution in public networks is driven by contextual triggers that set both the opportunities and constraints for change. These include *policy and funding cycles*, which define priorities, allocate resources, and establish the formal architecture of performance systems. *IT and reporting infrastructures* provide the technical means through which data are collected, validated, and transmitted, and shifts in these systems can alter what is measurable and comparable *External evaluations* introduce legitimacy pressures, identifying shortcomings and recommending reforms that may be taken up in subsequent cycles. Finally, *stakeholder feedback* (from service users, frontline organisations, oversight bodies, and funding agencies) supplies the continuous input necessary to question whether the PMS remains meaningful and proportionate. In any public sector network, these triggers act as external forces that periodically compel performance systems to adapt.

***Middle layer: the PMS lifecycle.*** Within these triggers, the PMS evolves through a patterned lifecycle that repeats across policy or funding cycles. The lifecycle does not involve continuous adjustment of indicators, but rather a phase-bounded process structured around four stages:

1. *Design*: At the beginning of a cycle, indicators, targets, and reporting obligations are specified, often through formal agreements, contracts, or regulations.
2. *Appropriation*: Once fixed, the PMS is enacted and internalised by network members. This involves training, capacity-building, peer learning, and the development of shared interpretations to ensure consistent application across diverse organisations.
3. *Evaluation*: Over time, the PMS is tested through monitoring, external reviews, and after-action assessments, generating evidence about its feasibility, credibility, and utility.
4. *Renewal*: At the end of the cycle, insights from evaluation and stakeholder feedback are synthesised into proposals for the next round of design. Renewal thus represents the moment when the PMS can be reshaped, but always within the context of new policy and funding priorities.

This lifecycle is transferable because most public sector networks operate under time-bound mandates or funding arrangements that naturally segment PMS development into distinct cycles, rather than allowing for continuous adjustment.

***Inner layer: building blocks.*** At the core of the framework are the building blocks that provide the technical and institutional foundation of the PMS. The *measurement logic* defines how the system conceptualises performance, for example by linking activities to outputs, outcomes, and broader impacts. *Contribution metrics* recognise that in networked settings, results are often co-produced and attribution is shared among multiple actors. The *efficencylens* introduces comparability and legitimacy, typically through ratios, benchmarks, or minimum standards. The *data pipeline* encompasses the definitions, evidence rules, IT systems, and audit protocols that ensure credibility and reproducibility of performance information. Finally, *governance arenas* (committees, working groups, or representative bodies) are the spaces where PMS are interpreted, contested, and redesigned. These building blocks are generic in the sense that any public sector network requires a performance logic, a way to attribute contributions, mechanisms for increasing efficiency, reliable data infrastructures, and institutional settings for governance.

***Integration of layers.*** The explanatory power of the framework lies in the interaction of the three layers. Contextual triggers generate the impetus for change, but they are translated into concrete adaptations only through the lifecycle, where design, appropriation, evaluation, and renewal unfold in sequence. The building blocks provide the stable components that enable each phase to function and give continuity across cycles. This configuration makes the framework transferable: regardless of policy domain, scale, or geography, public sector networks evolve their PMS by responding to contextual pressures, following bounded lifecycles, and relying on core building blocks.

This framework explains why PMS in networks are stable within funding cycles but evolve across them, and how technical building blocks interact with governance arenas to ensure both continuity and adaptability. More broadly, the framework demonstrates that PMS in networks should not be conceptualised as static indicator systems nor as continuously adaptable tools, but as episodic, socio-technical systems that balance contractual stability with responsiveness to external triggers and internal learning. By offering this layered and cyclical view, our study extends existing theory and opens new avenues for comparative research across domains such as health, education, innovation, and environmental governance.

## 5. Discussion

This study set out to answer the question: *How do performance management systems (PMS) evolve within public sector networks?* Drawing on a longitudinal case study of the Enterprise Europe Network (EEN), our findings indicate that PMS in such networks evolve through episodic cycles rather than continuous adjustment, with contractual boundaries delimiting periods of stability and opportunities for redesign. The generic three-layer framework developed (comprising contextual triggers, lifecycle phases, and building blocks) helps explain these dynamics and provides a transferable lens for examining PMS evolution across different policy domains. The framework ( Figure 3) links empirical findings to the broader research question by explaining how contextual dynamics and internal learning processes co-produce the system’s structural evolution. In this way, the framework captures both the adaptive and institutional dimensions of PMS change, aligning the discussion with the evidence presented in Table 2.

The findings show that the EEN’s PMS operates through iterative loops of design, appropriation, evaluation and renewal, in which performance information is continually re-interpreted and embedded into managerial practice. This demonstrates that the system functions as a *management* device as much as a *measurement* one: indicators do not merely record activity but guide reflection, coordination and learning across the Network. In this sense, the framework resonates with the distinction proposed by Bititci (2015) and Smith & Bititci (2017), confirming that the effectiveness of a PMS depends on how measurement results are used to stimulate dialogue and adaptive action.

The analytical process also shares affinities with the *bottom-up* PMS development logic described by Garengo and Biazzo (2012). Similar to their circular, participatory approach, the evolution of the EEN PMS emerged through cycles of local interpretation, evaluation and synthesis that progressively informed formal redesign. This parallel helps to rationalise the findings: both cases show that effective PMS innovation depends on iterative learning between operational practice and strategic intent, even though the EEN represents a large public-network rather than an individual-firm context.

This study contributes to the literature on PMS evolution in three interrelated ways, each of which extends and refines existing theoretical perspectives.

First, we reframe PMS evolution as episodic and contractually bounded. Much of the PMS literature has conceptualised performance systems as capable of continuous adaptation, with measures incrementally updated in response to strategic or operational shifts ( Bourne *et al.*, 2000; Braz *et al.*, 2011; Kennerley & Neely, 2002; Kennerley & Neely, 2003; Wouters & Sportel, 2005). This perspective is consistent with intra-organisational settings where managers retain discretion to recalibrate indicators in line with strategy. Our findings, however, show that in public sector networks such as the EEN, PMS are locked into multi-annual funding frameworks, with indicators fixed for the duration of contractual periods. Redesign occurs only at contractual boundaries, triggered by external evaluations, policy reforms, or IT infrastructure changes ( Agostino & Arnaboldi, 2018). This supports recent calls to pay greater attention to the temporal and institutional structuring of PMS evolution ( Waardenburg *et al.*, 2025) and suggests that models which assume linear, incremental change ( Hald & Mouritsen, 2018; van Helden *et al.*, 2012) must be extended to incorporate the episodic nature of networked governance systems.

Second, the study advances the institutional theory of PMS by emphasising networks as arenas of negotiated meaning. Prior research shows that PMS are not neutral tools but rather embody different institutional logics ( Andon *et al.*, 2007; Carlsson-Wall *et al.*, 2016; Quattrone & Hopper, 2001). Yet, few studies have examined how this plays out in transnational, multi-actor networks. In the EEN, PMS design and use required the reconciliation of bureaucratic logics of control, entrepreneurial logics of innovation, and community logics of trust and reciprocity ( Herranz, 2007; Heranz, 2010). Governance arenas, such as representative bodies, thematic groups, and time-bound KPI working groups, played a central role in mediating contestation and stabilising shared definitions of success. This resonates with Provan and Milward’s (2001) emphasis on negotiation in network performance, but also extends it by showing how PMS themselves become boundary objects ( Heranz, 2010; Millar *et al.*, 2001) that accommodate institutional diversity while ensuring comparability. The finding also connects to Vikstedt and Vakkuri (2025), who highlight the coexistence of multiple logics in collaborative PMS, and to Koppenjan (2008), who stresses that success in networks is always negotiated rather than objectively defined.

Third, the findings deepen socio-technical perspectives on PMS by foregrounding the role of infrastructures. PMS evolution is not solely a matter of indicator design or governance negotiation but also of infrastructural supports. Building on socio-technical systems theory ( Trist, 1981) and recent insights into digitalisation and big data integration in PMS ( Cosa & Torelli, 2024; Karina *et al.*, 2025), our analysis shows that CRM systems, reporting templates, metadata protocols, and audit procedures actively shape what counts as measurable and legitimate performance. In the EEN, the introduction of new IT systems or evidence protocols redefined both the scope of measurement and the comparability of results across consortia. This aligns with Moynihan’s (2008) argument that infrastructures not only enable performance information flows but also structure decision-making, and with Deschamps and Mattijs (2018), who underline the role of PMS in supporting organisational learning. By highlighting the infrastructural dimension, our study adds to recent work that urges scholars to view PMS as socio-technical artefacts embedded in political and institutional contexts ( Jansson *et al.*, 2020; Vikstedt & Vakkuri, 2025).

Taken together, these contributions extend theorisations of PMS in three directions: (i) by embedding temporality through episodic, contractually bounded cycles of change; (ii) by situating PMS as arenas of negotiated meaning where multiple institutional logics are reconciled; and (iii) by recognising infrastructural supports as constitutive elements of PMS evolution. This integrated perspective enriches performance management theory by moving beyond static models of indicator design and beyond purely technical accounts of system change, offering instead a socio-technical, institutional, and temporal understanding of how PMS evolve in public sector networks.

### Implications for policy and management

For policymakers, the analysis highlights that the design of PMS in networked programmes should be understood as a strategic policy instrument rather than a purely administrative device. In the case of the EEN, successive redesigns of the PMS have shaped not only what is measured but also how collaboration, accountability and learning are organised within the Network. The findings suggest that policy cycles, external evaluations, and IT infrastructures jointly act as levers through which public authorities steer collective behaviour and define what counts as valuable performance. Consequently, mandating outcome-oriented KPIs is necessary but insufficient: the effectiveness of such instruments depends on the institutional ecosystem in which indicators are interpreted and used. Policymakers should therefore invest in the creation of governance arenas, feedback mechanisms and data infrastructures that enable distributed actors to use performance information as a resource for improvement, not only for compliance.

The EEN experience also indicates that performance policy instruments can evolve to promote adaptability and alignment across programme cycles. By embedding evaluation and redesign into the PMS lifecycle, policymakers can create systems that remain responsive to shifting strategic priorities and operational realities. Such adaptability is valuable in other EU-supported networks, such as regional innovation, sustainability, or health ecosystems, where complex interdependencies make rigid indicator frameworks quickly obsolete.

For network managers, the study underscores that PMS implementation should be treated as an *ongoing design process* rather than a static reporting obligation. The middle layer of the framework ( Figure 3) shows that appropriation mechanisms (training, thematic groups, mentoring and quality assurance routines) are essential for transforming formal indicators into shared practices. Managers should view these processes as opportunities to develop a common understanding of definitions, evidence protocols and contribution metrics, ensuring that performance information supports coordination and learning across consortia. Investing in peer exchanges and collaborative review sessions allows the PMS to become a practical tool for dialogue and improvement rather than a compliance-driven reporting system.

The introduction of contribution metrics and efficiency ratios in the EEN provides a concrete model for incentivising collaboration and balanced efficiency in multi-actor settings. By linking achievement recognition to collective contribution and by contextualising outputs through ratios, the PMS encourages partners to pursue joint impact rather than isolated performance targets. Managers in other large-scale public programmes can adapt these mechanisms to strengthen fairness, comparability and motivation while preserving flexibility for contextual differences.

More broadly, the framework developed in this study provides actionable guidance for designing PMS in other policy-driven networks that face the dual challenge of ensuring comparability and respecting contextual diversity. The model suggests that sustainable performance governance requires aligning three layers: (1) policy and contextual triggers that define strategic expectations; (2) lifecycle mechanisms that embed iterative evaluation and redesign; and (3) structural building blocks (data systems, governance arenas and managerial routines) that institutionalise learning across cycles. For both policymakers and managers, recognising these interdependencies is key to transforming performance systems from bureaucratic artefacts into dynamic infrastructures for collective improvement and accountability.

### Limitations and venues for future research

Several limitations should be acknowledged. First, the study is based on a single-case design. While the EEN represents a revelatory case, further comparative research across different policy domains (e.g., health, environment, education) is needed to test the transferability of the proposed framework.

Second, the data sources rely heavily on archival and observational material from within the EEN. Although triangulation and reflexive strategies were applied, future research could complement this with SME-level perspectives on how performance indicators influence service uptake and perceived value.

Third, the study focuses on the formal PMS as codified in EU calls, evaluations, and reporting tools. Informal practices of sensemaking, negotiation, and local adaptation deserve closer ethnographic attention to capture how PMS are enacted “on the ground” ( Andon *et al.*, 2007).

Future research could therefore pursue three directions: (i) cross-case comparisons of PMS evolution in different transnational networks; (ii) mixed-methods studies that integrate performance data with qualitative accounts of user experiences, including informal practices. The third direction could be the analysis of digitalisation and big-data integration in network PMS, extending socio-technical perspectives to account for emerging data sources and analytics ( Cosa & Torelli, 2024; Karina *et al.*, 2025).

## 6. Conclusion

This paper examined how PMS evolve in public sector networks through a longitudinal case study of the EEN. By tracing fifteen years of PMS design, appropriation, evaluation, and renewal, it showed that evolution is not linear or continuous but episodic and contractually bounded, shaped by contextual triggers and enacted through governance arenas.

The proposed three-layer framework (contextual triggers, lifecycle phases, and building blocks) offers a transferable model for understanding PMS evolution in networks. It demonstrates that PMS should be seen as socio-technical systems that balance stability and adaptability, integrating measurement logic, contribution metrics, efficiency lenses, data infrastructures, and governance arenas.

The findings contribute to theory by embedding temporal, institutional, and infrastructural dimensions into PMS research, and to practice by providing actionable lessons for policymakers and network managers seeking to design performance systems that foster both accountability and collaboration.

Ultimately, by recognising PMS as evolving design processes rather than static indicator sets, public sector networks can better capture the value of collaborative action and support SMEs — and other beneficiaries — in addressing Europe’s most pressing economic and societal challenges.

## Ethics and consent

This study is based on archival materials and researcher memos. The memos draw on the researcher’s participation in internal meetings, working groups, and training sessions within the Enterprise Europe Network. No personal data, sensitive information, or commercially confidential material are included in this study. All evidence is presented in aggregate or anonymised form, in line with organisational guidelines and ethical best practices. As the research relies on documentary sources and non-identifiable observational material, it did not require formal ethical approval or written informed consent from individual participants.

## Data Availability

Zenodo: Underlying data - PMS of the EEN ( https://doi.org/10.5281/zenodo.16911874) Ruiz-Coupeau (2025). This research contains the following underlying data: Call for proposals CELEX_C2006_306_07_EN Terms of reference EEN 2017–2018_en Call for proposals_smp-cosme-2021-een_en Call for proposals_smp-cosme-2024-een_en Evaluation report_eip_en 2011 Evaluation report_eaci_2011_en Evaluation report_EEN_2008–2014 Memo PMS used by the EEN period 2008–2025 Memo CESEAND Plus Period 2015–2016 Memo CESEAND Plus Period 2019–21 Memo Working Group on Measurement and KPIs Memo Training Improve Your Achievements Memo Thematic Group Start-up and Scale-up Conference proceedings on EEN (2008–2020) Report EEN Our Story 2008–2020 Data are available under the terms of the Creative Commons Attribution 4.0 International license (CC-BY 4.0)

## Footnotes

^1^ Enterprise Europe Network https://een.ec.europa.eu/

^2^ SME Performance Review 2023, https://single-market-economy.ec.europa.eu/smes/sme-strategy/smeperformance-review_en

^3^ SME Strategy for a sustainable and digital Europe, COM(2020) 103 final.

^4^ A New Industrial Strategy for Europe, COM(2020) 102 final.

^5^ SME Relief Package, COM(2023) 535

^6^ European Commission’s Single Market Programme https://commission.europa.eu/funding-tenders/find-funding/eu-funding-programmes/single-market-programme/overview_en

^7^ European Innovation Council and SMEs Executive Agency (EISMEA)

^8^ CESEAND – Enterprise Europe Network node of the region of Andalucia (Spain) https://een-andalucia.es/

^9^ European Commision’s Competitiveness and Innovation Programme https://ec.europa.eu/cip/

## Figure permissions

The authors have created and own Figure 3. Figure 1 and Figure 2 were obtained from EU Funding & Tenders Portal. And as per the European Commission’s publications rules, these are licensed under the Creative Commons Attribution 4.0 International (CC BY 4.0) licence, which implies that they can be reused, if the source is acknowledged. That has been done in this manuscript. More information could be found here https://commission.europa.eu/legal-notice_en#copyright-notice
