# Supplementary material for: Evolving Performance Management Systems in Public Sector Networks: A Longitudinal Case Study of The World Largest Public Network Supporting Smes
Source: Open Res Eur. 2026 Jan 8;5:286. Originally published 2025 Sep 19. [Version 2] doi: 10.12688/openreseurope.21281.2 (PMC12789862; doi:10.12688/openreseurope.21281.2)
Supplement: Peer review history

# Reviewer report: Reviewer response for version 2

**DOI:** 10.21956/openreseurope.24029.r67776

**Published:** 2026-01-28

**Authors:** Aylin Ates

**Affiliations:** 1 University of Strathclyde, Glasgow, Scotland, UK

The authors have satisfactorily addressed my comments in the revised manuscript. I have no further concerns and recommend the article for indexing.

If applicable, is the statistical analysis and its interpretation appropriate?

Not applicable

Is the study design appropriate and is the work technically sound?

Yes

Is the work clearly and accurately presented and does it engage with the current literature?

Yes

Are the conclusions drawn adequately supported by the results?

Partly

Are sufficient details of methods and analysis provided to allow replication by others?

Partly

Are all the source data and materials underlying the results available?

Partly

Reviewer Expertise:

performance management, strategic management, SMEs, manufacturing ecosystems, digital transformation, innovation

I confirm that I have read this submission and believe that I have an appropriate level of expertise to confirm that it is of an acceptable scientific standard.

# Reviewer report: Reviewer response for version 2

**DOI:** 10.21956/openreseurope.24029.r67775

**Published:** 2026-01-12

**Authors:** Alberto Sardi

**Affiliations:** 1 Department of Management, University of Turin, Turin,, Italy

Dear Authors,

 Congratulations on your improvements. I am pleased to accept your manuscript in its current form.

 Sincerely

If applicable, is the statistical analysis and its interpretation appropriate?

Not applicable

Is the study design appropriate and is the work technically sound?

Yes

Is the work clearly and accurately presented and does it engage with the current literature?

Yes

Are the conclusions drawn adequately supported by the results?

Partly

Are sufficient details of methods and analysis provided to allow replication by others?

Partly

Are all the source data and materials underlying the results available?

Partly

Reviewer Expertise:

Performance measurement, Performance management, Business performance

I confirm that I have read this submission and believe that I have an appropriate level of expertise to confirm that it is of an acceptable scientific standard.

# Reviewer report: Reviewer response for version 2

**DOI:** 10.21956/openreseurope.24029.r67774

**Published:** 2026-01-10

**Authors:** G. Jan van Helden

**Affiliations:** 1 University of Groningen, Netherlands, Groningen, The Netherlands

I saw how authors have accommodated my review and I am happy with revisions.

If applicable, is the statistical analysis and its interpretation appropriate?

Not applicable

Is the study design appropriate and is the work technically sound?

Yes

Is the work clearly and accurately presented and does it engage with the current literature?

Yes

Are the conclusions drawn adequately supported by the results?

Yes

Are sufficient details of methods and analysis provided to allow replication by others?

Partly

Are all the source data and materials underlying the results available?

Partly

Reviewer Expertise:

NA

I confirm that I have read this submission and believe that I have an appropriate level of expertise to confirm that it is of an acceptable scientific standard.

# Reviewer report: Reviewer response for version 1

**DOI:** 10.21956/openreseurope.23017.r64013

**Published:** 2025-12-10

**Authors:** G. Jan van Helden

**Affiliations:** 1 University of Groningen, Netherlands, Groningen, The Netherlands

I enjoyed reading your paper. It discusses an interesting theme in an eloquent way. Moreover, your paper is well-structured, and your arguments are clearly presented.

 Below are some reflections that might inspire you to further improve your paper.

 1. The research question is very descriptive, as it is formulated as follows; How do performance management systems in public sector networks evolve?

 Because of your literature review, I would have expected a more specific research question that is engaged with the literature and gives guidance to your data analysis. 

 Some clues might be helpful.

 First, a network comprises different actors with diverging tasks and responsibilities. In terms of the PMS it is appropriate to investigate how performance indicators are associated with these diversity of actors and the network as a whole.

 Second, the dynamics of a PMS may be contingent upon changes in the governance of the network over time,  and partly interrelated with changes in the environment of the network, such as economic and technological developments in small and medium-size businesses. 

 Third, the PMS is potentially impacted by power dynamics, in the sense of actors becoming less or more powerful over time.

 My suggestion is to think about a framework in which these factors are positioned. This framework can  also be helpful in articulating the contributions to existing knowledge your study makes.

 Please mind that these are just suggestions. It is up to you to adopt them or replace them for better ones.

 See also point 5 below.

 2. The European Enterprise network (EEN) is the case study in this paper. Without further clarification I have doubts about the question whether this is a really interesting network with different actors at various layers. It seems to be an organization with 60 members in various countries, so it is manly an association. What are here the actors: the individual members, the coordinating institutions of EEN, or the representatives of the EU commission. This is all unclear. I am familiar with network PMSs for transportation (train companies, bus companies, governing governments and funding governments), and with the asylum facilitating networks (admission, selection, housing, etc.). Please clarify this.

 3. Your data analysis as introduced in section 4 is sophisticated.  This is a strong point of your study. One issue came to my mind when reading about the types of data sources. What I missed was interviews with key actors for unravelling motives behind decisions made about the PMS  or are the studied notes of the network sufficiently informative in this respect? Please clarify.

 4. Your findings section is interesting with a clear division between the various phases in the development of the PMS. It was not always clear to me why a PMS was revised from one stage to the other. Maybe you can give a bit more explanation.

 5. The paper culminates in the conceptualisation depicted in figure 3. This is an impressive conceptualization with a layered structure, including contextual triggers, a PMS life cycle, and inner-layer building blocks. Although I find this figure impressive, it is not extensively clarified in the paper how the study of underlying documents leads to these outcomes. It looks a bit as a consultancy product: nice to know but not always clear how it can be justified by relevant data sources. The paper claims to use an abduction approach of its data analysis, which is meant to find likely explanations for empirical phenomena, but the data analysis largely remains a black box.

If applicable, is the statistical analysis and its interpretation appropriate?

Not applicable

Is the study design appropriate and is the work technically sound?

Yes

Is the work clearly and accurately presented and does it engage with the current literature?

Yes

Are the conclusions drawn adequately supported by the results?

Yes

Are sufficient details of methods and analysis provided to allow replication by others?

Partly

Are all the source data and materials underlying the results available?

Partly

Reviewer Expertise:

Publici sector accounting, public sector performance measurement, public management

I confirm that I have read this submission and believe that I have an appropriate level of expertise to confirm that it is of an acceptable scientific standard, however I have significant reservations, as outlined above.

# Response

**Published:** 2025-12-15

**Authors:** Stéphane Ruiz Coupeau

**Affiliations:** Universidad de Sevilla, Seville, Andalusia, Spain

We thank Reviewer 3 for their positive evaluation of the manuscript and for the constructive reflections offered, which helped us clarify several elements of the study. Below we address each point in turn.

 1. Research question We acknowledge the reviewer’s observation regarding the descriptive nature of the research question. In this study, the formulation was intentional, as the abductive, exploratory design seeks to reconstruct underlying mechanisms of PMS evolution in complex public networks rather than test predefined hypotheses. Following the reviewer’s suggestion, we strengthened the link between the research question, the literature review and the analytical framework, clarifying how the three-layer structure (contextual triggers, lifecycle phases, building blocks) provides theoretical and empirical specificity to the question without altering its exploratory form. Given the abductive purpose, we consider the current formulation appropriate.

 2. Nature of the Enterprise Europe Network (EEN) We thank the reviewer for raising this important point. To avoid misunderstanding, we clarified in Section 3 that the EEN is not a small association but a large public network of nearly 600 regional partner organisations across Europe. It is structured through multi-level governance, including the European Commission/EISMEA, national and regional consortia, and cross-cutting governance bodies such as the Representative Body and thematic groups. These actors hold differentiated roles within the network. A short clarification has been added to the manuscripts and responsibilities to make this explicit.

 3. Data sources and absence of interviews We appreciate the reviewer’s point regarding motives behind PMS decisions. The primary material used in this study includes research-generated memos from governance arenas such as KPI Working Groups, Steering Committees, Technical Boards and thematic groups settings in which PMS definitions, interpretations and redesign decisions are explicitly debated. These sources therefore provide rich access to actors’ underlying motives and sensemaking processes. While interviews could have added complementary perspectives, we believe the empirical corpus is sufficiently robust for the study’s objectives. A brief clarification of this rationale has been added to Section 3.

 4. Explanation of PMS transitions between phases We thank the reviewer for this observation. The revised manuscript already distinguishes between the empirical description of PMS phases (Section 4.1) and the possible explanation of the factors that account for transitions between these phases (Section 4.2). To make this relationship more visible, we added a short bridging sentence indicating that the generic framework captures precisely the triggers and mechanisms that account for phase shifts.

 5. Conceptualization in Figure 3 and link to data We appreciate the reviewer’s thoughtful comment. In response, we expanded Table 2 to present the empirical foundations and coding progression from open codes to axial and selective categories. This addition clarifies how the conceptualisation in Figure 3 emerges from the underlying data and strengthens transparency in the abductive reasoning process. Together with the expanded explanation in Section 4.2, we believe the link between the documentary evidence, coding process and the resulting framework is now fully traceable.

# Reviewer report: Reviewer response for version 1

**DOI:** 10.21956/openreseurope.23017.r63284

**Published:** 2025-11-20

**Authors:** Alberto Sardi

**Affiliations:** 1 Department of Management, University of Turin, Turin,, Italy

The paper is of considerable interest as it addresses several fundamental aspects of performance measurement. It investigates performance management systems within public sector networks through a longitudinal single-case analysis of the Enterprise Europe Network (EEN), a pan-European network supporting SMEs. The academic literature still shows some gaps on this topic, mainly due to the inherent complexity of conducting research in such large and multifaceted networks.

 The research design is well-structured and carefully organized, as is the literature review. However, the theoretical framework and reference models could be further developed to strengthen the discussion and enhance the potential contributions of the study.

 Given the complexity of the case under investigation, the findings sometimes appear mainly descriptive or based on the authors’ insights and experience. While this adds value in terms of contextual understanding, it may partially limit the scientific robustness of the work, particularly regarding its generalizability and replicability.

 To further improve the study, it would be beneficial to refine some methodological aspects, especially those related to data collection and data analysis. The sources of primary data and the procedures adopted for data gathering could be presented in a more detailed and structured way (e.g., specifying the use of documents, memos, field notes, and observations made during meetings). The data analysis process would also benefit from a more comprehensive explanation to increase methodological transparency and support replicability.

 Two final considerations may contribute to further improvement.

 First, regarding the topic of the study, in my view the analysis focuses primarily on the performance measurement  – that is, on what to measure – rather than on the performance management, which deals with how to use measurement results (Bititci, 2015; Smith & Bititci, 2017).

 Second, the approach adopted appears to be similar to the bottom-up approach proposed by Garengo and Biazzo, particularly in the first two stages described in “Unveiling strategy in SMEs through balanced scorecard implementation: A circular methodology.” Referring to this work could help the authors better rationalize and interpret their findings.

 Overall, I congratulate the authors for this interesting and valuable work.

If applicable, is the statistical analysis and its interpretation appropriate?

Not applicable

Is the study design appropriate and is the work technically sound?

Yes

Is the work clearly and accurately presented and does it engage with the current literature?

Yes

Are the conclusions drawn adequately supported by the results?

Partly

Are sufficient details of methods and analysis provided to allow replication by others?

Partly

Are all the source data and materials underlying the results available?

Partly

Reviewer Expertise:

Performance measurement, Performance management, Business performance

I confirm that I have read this submission and believe that I have an appropriate level of expertise to confirm that it is of an acceptable scientific standard, however I have significant reservations, as outlined above.

# Response

**Published:** 2025-12-12

**Authors:** Stéphane Ruiz Coupeau

**Affiliations:** Universidad de Sevilla, Seville, Andalusia, Spain

We thank Reviewer 2 for their insightful comments and positive assessment of the manuscript.

 In response to the suggestion to further develop the theoretical framework, the revised version expands the discussion of key concepts in performance measurement and performance management, incorporating contributions from Bititci (2015) and Smith and Bititci (2017).

 The paper also now acknowledges and integrates parallels with bottom-up PMS development approaches, including Garengo and Biazzo’s circular methodology, which helps contextualise and deepen the interpretation of the observed redesign processes.

 To address concerns about the descriptive nature of the findings and the need for clearer methodological transparency, Section 3 now provides a more detailed account of data collection procedures and the composition of the primary qualitative material.

 The revised manuscript specifies how memos, field notes and archival sources were generated and used, clarifies the structure of the qualitative data corpus and explains how the longitudinal nature of the case is grounded in these materials.

 Section 4 and Table 2 now incorporate additional empirical excerpts, making the evidence-interpretation link more visible and addressing the reviewer’s concern regarding robustness and replicability.

 Finally, the revised Discussion clarifies more directly how PMS in the Network evolve not only through shifts in what is measured but through managerial processes of appropriation, coordination and evaluation. This helps rebalance the analysis toward the “management” dimension highlighted by the reviewer.

 Overall, the revised manuscript strengthens its theoretical grounding, methodological transparency and empirical substantiation, directly addressing the points raised by Reviewer 2 while preserving the integrity of the longitudinal case design.

# Reviewer report: Reviewer response for version 1

**DOI:** 10.21956/openreseurope.23017.r61554

**Published:** 2025-10-20

**Authors:** Aylin Ates

**Affiliations:** 1 University of Strathclyde, Glasgow, Scotland, UK

The paper aims to conduct a longitudinal single case study of the Enterprise Europe Network (EEN), a pan-European SME support network, to answer the research question: h *ow do performance management systems evolve within public sector networks?*

 The paper adopts a qualitative research design using mostly secondary data sources supported by primary data following an ethnography type research. The topic of the paper is relevant and significant. It is well written in general. However, the paper also has some shortcomings that should be addressed in a revision:

 - The literature review is well organised and critical. Research gaps and problems in practice are presented clearly, which is very good. Indeed, we need further research into performance management in networks, ecosystems and inter-organisational settings, which are increasingly essential to make collaborative innovation and transformation happen. 

 - **Data sources:** The primary data sources must be clarified. The authors claim to use memos, field notes and observations in various meetings. However, this type of data is not sufficiently referenced or used to support the findings.

 - **Qualitative data analysis**: Data analysis is explained regarding the coding exercise, but more evidence should be provided to support the key findings. For example, direct quotes from the interviews/ meetings or short illustrative examples from direct observations can be used to provide further evidence for the findings. Also, inter-coder reliability practices should be explained. Who conducted the data analysis? Only one researcher? Or multiple researchers? 

 - **Contributions:** The Figure 3 does not show the evolutionary phases with links to the contextual triggers in a convincing way. This framework can be developed further that addresses the research question and reflects the Discussions better. Second, implications for policy should be expanded as this section is very brief in the current paper. 

 Finally, good luck with developing this work further.

If applicable, is the statistical analysis and its interpretation appropriate?

Not applicable

Is the study design appropriate and is the work technically sound?

Yes

Is the work clearly and accurately presented and does it engage with the current literature?

Yes

Are the conclusions drawn adequately supported by the results?

Partly

Are sufficient details of methods and analysis provided to allow replication by others?

Partly

Are all the source data and materials underlying the results available?

Partly

Reviewer Expertise:

performance management, strategic management, SMEs, manufacturing ecosystems, digital transformation, innovation

I confirm that I have read this submission and believe that I have an appropriate level of expertise to confirm that it is of an acceptable scientific standard, however I have significant reservations, as outlined above.

# Response

**Published:** 2025-12-12

**Authors:** Stéphane Ruiz Coupeau

**Affiliations:** Universidad de Sevilla, Seville, Andalusia, Spain

We thank Reviewer 1 for their constructive feedback.

 The revised manuscript now clarifies and strengthens the primary data foundations of the study by explaining more explicitly how researcher memos and archival documentation were generated and used.

 Section 3 distinguishes primary from secondary sources and indicates that memos and field notes from governance meetings, working groups and training events constitute core evidence supporting the findings.

 Section 4 and Table 2 now include concrete empirical excerpts that illustrate partners’ discussions, evaluation feedback and interpretive tensions, thereby making the connection between raw data and analytical categories more explicit.

 The methodological section has been expanded to explain the qualitative analysis more clearly. It now specifies how open, axial and selective coding were conducted by the first author and how the co-authors reviewed coding structures and category definitions to strengthen analytical robustness.

 This addresses the reviewer’s question regarding inter-coder reliability and the role of multiple researchers. The generic framework has been refined to present more convincingly the links between contextual triggers, the PMS lifecycle and the building blocks.

 Figure 3 itself has been refined to depict these three layers and their interrelations more clearly, with arrows indicating how external triggers feed into lifecycle phases and how these phases reshape the building blocks over successive cycles.

 The Discussion section articulates more explicitly how this framework answers the research question. Finally, the implications for policy and management have been substantially expanded. The revised manuscript discusses how policymakers can use PMS design to steer network behaviour, how governance arenas support learning and coordination, and how managers can foster appropriation and balanced efficiency across consortia.

 Overall, the revised paper offers clearer methodological transparency, stronger empirical grounding and a more fully developed contribution that directly addresses Reviewer 1’s concerns.
